# A study on the genetic comorbidity between autoimmune diseases and pulmonary hypertension: an observational study and POST-GWAS analysis

**DOI:** 10.7150/ijms.107884

**Published:** 2025-02-18

**Authors:** Biao Hu, Haoyu Zhong, Raymond Shi, Zeru Chen, Aofeng Liu, Xiang Li, Dansha Zhou, Jiaxuan Lai, Chenting Zhang, Yuqin Chen, Jian Wang

**Affiliations:** 1 State Key Laboratory of Respiratory Diseases, National Center for Respiratory Medicine, National Clinical Research Center for Respiratory Diseases, Guangdong Key Laboratory of Vascular Diseases, Guangzhou Institute of Respiratory Health, the First Affiliated Hospital of Guangzhou Medical University, Guangzhou, Guangdong, China, 510120.; 2 Guangzhou Medical University, Guangzhou, Guangdong, China, 510260.; 3 International Department, The Affiliated High School of South China Normal University, Guangzhou, Guangdong, China, 510631.; 4 Guangzhou Laboratory, Guangzhou International Bio Island, Guangzhou, Guangdong, China, 510320.

**Keywords:** pulmonary hypertension, autoimmune diseases, shared genetic architecture, Mendelian randomization

## Abstract

**Background:** The causal relationship between various prevalent autoimmune diseases (ADs) and pulmonary hypertension (PH) has yet to be fully understood, and the contribution of genetic factors to their coexistence remains largely unexplored.

**Methods:** We utilized Post-GWAS and the Medical Information Mart for Intensive Care (MIMIC) database to investigate the relationship between autoimmune diseases and PH.

**Results:** After a series of MR Analyses, only Type 1 diabetes Mellitus (T1DM) (OR = 1.06, 95% CI 0.99-1.13, *P* = 0.083; OR = 1.07, 95% CI 1.02-1.13, *P* = 0.005) and primary biliary cholangitis (PBC) (OR = 1.10, 95% CI 1.05-1.15,* P* = 8.22E-5; OR = 1.08, 95% CI 1.03-1.14, *P* = 0.002) emerged as significantly correlated with PH. Additionally, reverse MR indicated that PH could trigger the development of systemic lupus erythematosus (SLE) (OR=1.090, 95% CI = 1.014-1.171, *P* = 0.014). An observational study using real-world data found a clear association between rheumatoid arthritis and increased risk of PH after adjusting confounding various variables (OR = 1.39, 95% CI 1.11-1.75, *P* = 0.005). Furthermore, the genetic correlation results between the diseases: T1DM & PAH: *P*_(LDSC)_ = 1.20e-11, *P*_(GNOVA)_ = 3.36e-08; PBC & PAH: *P*(_LDSC)_ = 9.40e-07, *P*_(GNOVA)_ = 5.17e-05.

**Conclusions:** Our study indicates a genetic correlation and shared risk genes between PH and autoimmune diseases, offering insights into the mechanisms underlying their co-occurrence and potential implications for future therapeutic strategies.

## Introduction

Pulmonary hypertension (PH) is a syndrome defined by a resting mean pulmonary artery pressure (mPAP) of 20 mm Hg or above^1^. Clinically, PH is categorized into five main classes: pulmonary arterial hypertension (PAH), PH resulting from left heart disease, PH owing to lung diseases and/or hypoxia, chronic thromboembolic PH, and PH of uncertain multifactorial mechanisms^2^. PAH is classified as Group I PH, representing a rare pulmonary small vessel disease characterized by vascular damage, endothelial dysfunction, proliferation, and remodeling^3^. There are an estimated 5.6-25 cases of PAH per million adults^4^, compared to 4.8-8.1 cases per million children^5^. Despite advancements in the treatment of PAH, the prognosis remains bleak with a high mortality rate^6^, ^7^.

Autoimmune diseases (ADs) refer to a collection of diseases brought on by the immune system becoming less tolerant to autoantigens and by improper immune system activation, which leads to the production of autoantibodies and immunological-mediated tissue damage^8^, ^9^. Research has illuminated a profound connection between Systemic Lupus Erythematosus (SLE) and PH^10^, with the overall mortality rate for PH patients admitted to Intensive Care Units (ICU) exceeding 27%^11^, ^12^. Furthermore, conditions like Type 1 Diabetes Mellitus (T1DM), Primary Biliary Cholangitis (PBC) and Rheumatoid Arthritis (RA) are also implicated in the etiology of PH, displaying notable gender disparities^13^. Notably, thyroid dysfunction has been identified as a potential risk factor for PH^14^-^16^, and autoimmune thyroid diseases (AITD), such as Graves' disease and Hashimoto's thyroiditis, are thought to be potential modulators of PH development^17^, ^18^. It is quite evident that the link between ADs and PH has always been a focal point of interest, especially considering the pivotal role that inflammation and immune responses triggered by ADs play in the pathogenesis of PH^19^. Specially, chronic inflammation associated with ADs not only results in abnormal proliferation of smooth muscle cells in the pulmonary arteries, which leads to vascular remodelling^20^, but also damages endothelial cells and causes pulmonary vasoconstriction, which results in PH^19^, ^21^.

Nevertheless, as a result of the intrinsic limitations of conventional observational study designs, current observational studies cannot entirely eliminate the likelihood of reverse causality and confounding variables, which could introduce prejudiced associations and conclusions^22^, ^23^. Hence, MR analysis was utilized in this study to assess the relationship between genetically predicted ADs and PH.

**Figure 1:**
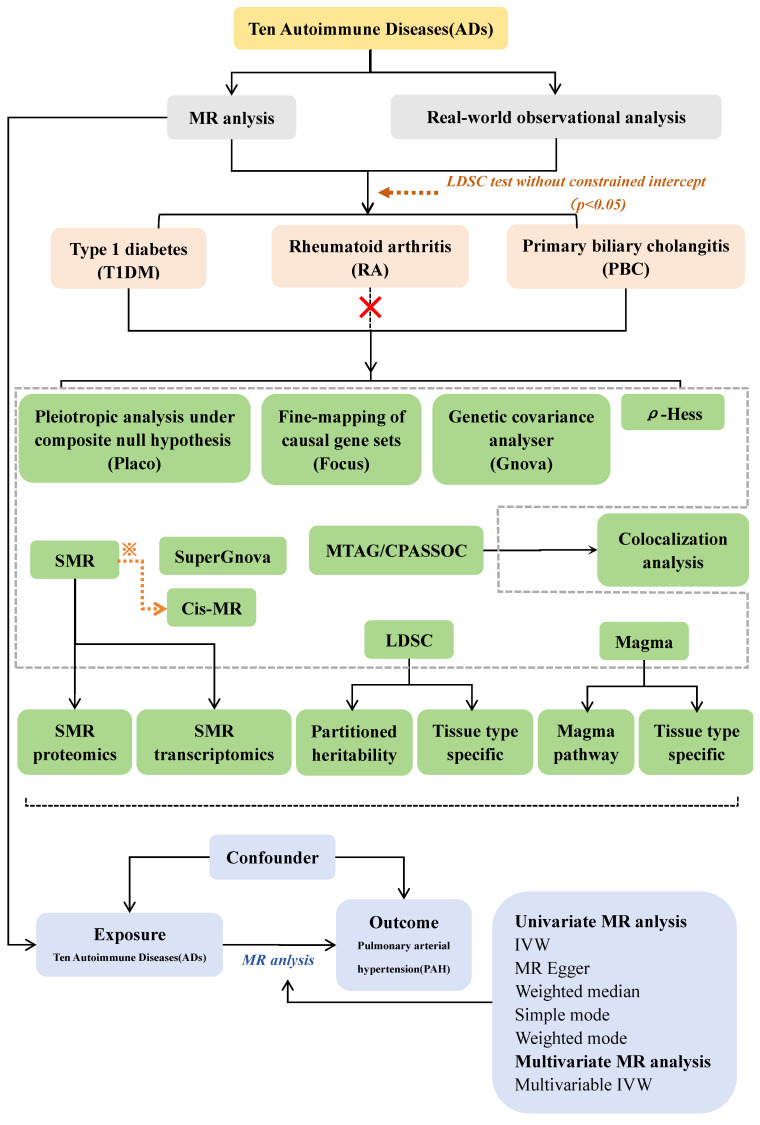
** Study workflow. ※:** To be selected as candidate genes for cis-MR validation, both autoimmune diseases and pulmonary arterial hypertension must satisfy the following criteria: P_(SMR)_ < 0.05, P_(HEDI)_ > 0.05, and Total loci ≥ 2.

Here, using large-scale genome-wide association study (GWAS) summary statistics Mendelian randomization (MR) study and GWAS post analysis which in **Figure.1**.

## Methods

### Mendelian randomization

#### Study design

**Figure 1** illustrates our study design and MR Hypothesis. The objective of this research endeavor was to examine the causal connection that exists between 10 ADs and PAH. MR was implemented in accordance with three hypotheses^24^. Assumption 1: Instrumental variables (IVs) are strongly correlated with exposure; Assumption 2: IVs are independent of potentially confounding variables; and Assumption 3: IVs only affect outcomes through exposure, not through any other means. In addition, we adhered to the recommendations of STROBE-MR 25 to guarantee the transparency and reproducibility of our study.

#### Data sources and instruments selection

The GWAS summary statistics of ADs and PAH were obtained from the FinnGen Consortium (R9) (https://www.finngen.fi/fi), the MRC-IEU database (https://gwas.mrcieu.ac.uk/) and the MRC-IEU database (https://gwas.mrcieu.ac.uk/).

To investigate the potential mechanisms underlying the genetic link between ADs and PAH, we further calculated 21 potential mediators for analysis. The specifics of all data sources can be found in **Supplemental Tables for MR 1 and 2**.

To fulfil the three previously mentioned stringent assumptions, we conducted a series of quality control procedures to identify suitable single nucleotide polymorphisms (SNPs). The detailed procedure we have supplemented in **Supplemental Tables for MR1**.

#### Statistical analyses

We employed the Inverse Variance Weighted (IVW) for univariate, multivariate, and reverse MR analyses. Additionally, a two-step mediation MR analysis was conducted to ascertain whether the relationship is mediated by potential intermediate factors, the specifics of all statistical analyses can be found in **Supplemental Tables for MR1 and MR2**. All statistical analyses were conducted utilizing Mendelian Randomization (0.4.2), TwoSampleMR (0.5.7), MRPRESSO (1.0), and MVMR (0.3) in R version 4.2.2.

### Real-world observational analysis

#### Data sources

This study conducted a retrospective cohort analysis using data from the Medical Information Mart for Intensive Care-IV (MIMIC-IV) (version 1.0)^26^.

#### Population

Our main focus is on patients diagnosed with PH upon admission. The methods used to diagnose the disease and the data included in our observational study are described in the *Supplemental* *Methods for MR and cross-sectional study* of Supplementary Material**.**

#### Statistical analyses

Patients were divided into PH and non-PH groups according to whether PH occurred or not. For the detailed statistical analysis methods during the analysis, we have given a detailed introduction in *Supplemental* *Methods for MR and cross-sectional study* of Supplementary Material. The analysis was carried out using R software version 4.1.3 and SPSS version 22.0 (IBM SPSS Statistics, Armonk, NY, USA). A p-value below 0.05 was considered statistically significant.

### Post-GWAS analysis

We have given a detailed introduction in *Supplemental Methods for Post-Gwas analysis* of Supplementary Material.

#### LDSC and SUPERGNOVA

##### Study design

We employ linkage disequilibrium score regression (LDSC) as a crucial method for estimating genetic correlations across multiple traits or diseases^27^, ^28^.

### MAGMA

#### Study design

As a sensitivity analysis for LDSC, we conducted tissue-specific enrichment and gene set analyses using Multimarker Analysis of GenoMic Annotation (MAGMA)^29^.

#### Local genetic correlation analysis

##### ρ-HESS

Heritability estimation using Heritability Estimation from Summary Statistics(ρ-HESS) is a method employed to estimate local SNP heritability and genetic correlations^30^. To account for multiple comparisons, we applied a Bonferroni correction (0.05/ the number of regions) to ensure statistical rigor^30^.

#### Colocalization analysis

We utilized the coloc.abf function from the coloc R package (version 5.1.0) with its default prior to perform colocalization analysis (COLOC).

#### PLACO: pleiotropic analysis under composite null hypothesis

We initially extracted disease data relevant to sets of paired trait alliances exhibiting notable genetic correlations or overlap from the GWAS database. Subsequently, we employed polytomy analysis under the composite null hypothesis (PLACO)^31^ to identify potential polytomous single-nucleotide variants (SNVs). SNVs with a PLACO *FDR*-value < 5 × 10^-8^ were deemed statistically significant.

#### Summary-data-based Mendelian randomization (SMR) and cis-MR

We employed SMR to identify potential functional genes implicated in the statistical associations of PAH and ADs^32^. To be selected as candidate genes for cis-MR [**Figure 10**] validation, both ADs and PAH must satisfy the following criteria: *P*_(SMR)_ < 0.05, *P*_(HEDI)_ > 0.05, and Total loci ≥ 2[**Figure 9**].

#### FOCUS

We employed fine-mapping of causal gene sets (FOCUS) to compute the posterior inclusion probability (PIP) for each gene within significant bivariate loci^33^.

## Results

### Mendelian randomization

#### Genetic instruments

For forward MR, the F-statistics of the chosen independent SNPs ranged from 20 to 1480, suggesting that these instrumental variables are unlikely to exhibit weak instrumental bias, as demonstrated in **Table S3** and **Table S4**. In reverse MR, after a series of SNP screening steps, a total of 20 PAH-related SNPs were eligible for IVs, and the F-statistics of these IVs were all greater than the threshold of 10, details are provided in **Table S14**.

#### Univariate MR analysis

We performed MR analyses of 15 ADs using five different methods in two different sources of pooled data, and then performed comparative analyses of MR results for the same ADs (**Table S5 and Figure S1**). IVW analysis revealed significant and causal associations between genetically predicted Celiac disease ( OR = 1.071, 95% CI 1.007-1.138, *P* = 0.028; OR = 1.133, 95% CI 1.017-1.263, *P* = 0.024), PBC (OR = 1.144, 95% CI 1.071-1.222, *P* = 7.06E-5; OR = 1.100, 95% CI 1.034-1.171, *P* = 2.50E-3) , T1DM (OR = 1.250, 95% CI 1.021-1.531, *P* = 0.031; OR = 1.139, 95% CI 1.048-1.238, *P* = 0.002), Graves' Disease (OR = 1.121, 95% CI 1.006-1.249, P = 0.038), and Hashimoto's Thyroiditis (OR = 1.255, 95% CI 1.043—1.511, P = 0.016) with PAH. These conditions were found to increase the risk of developing PAH. Of note, genetically predicted genetic associations between ankylosing spondylitis and PAH differed significantly between two pooled databases (OR = 0.941, 95% CI 0.888-0.997, *P* = 0.040; OR = 0.958, 95% CI 0.567-1.618, *P* =0.871), suggesting a potential causal relationship between the two conditions, and ankylosing spondylitis possibly reduces the risk of PAH. There was no notable genetic correlation found between other ADs (CD, MS, Psoriasis, SLE, UC, Mixed connective tissue disease, Sjogren syndrome, and Systemic sclerosis) and PAH. Also, we further analyzed the relationship between IVs containing MHC loci associated with ADs and PAH, as demonstrated in **Figure S2**. Surprisingly, the results of MR analysis containing MHC loci remained consistent with those of MR analysis without MHC loci.

In the sensitivity analysis, this study assessed heterogeneity and pleiotropy, respectively, and the corresponding results are presented in **Table S5** and **Table S6**. The *P*-value for each ADs was also higher than 0.05, indicating that the results of the MR analyses were not affected by heterogeneity and pleiotropy.

#### Multivariate MR analysis

**Figure 2:**
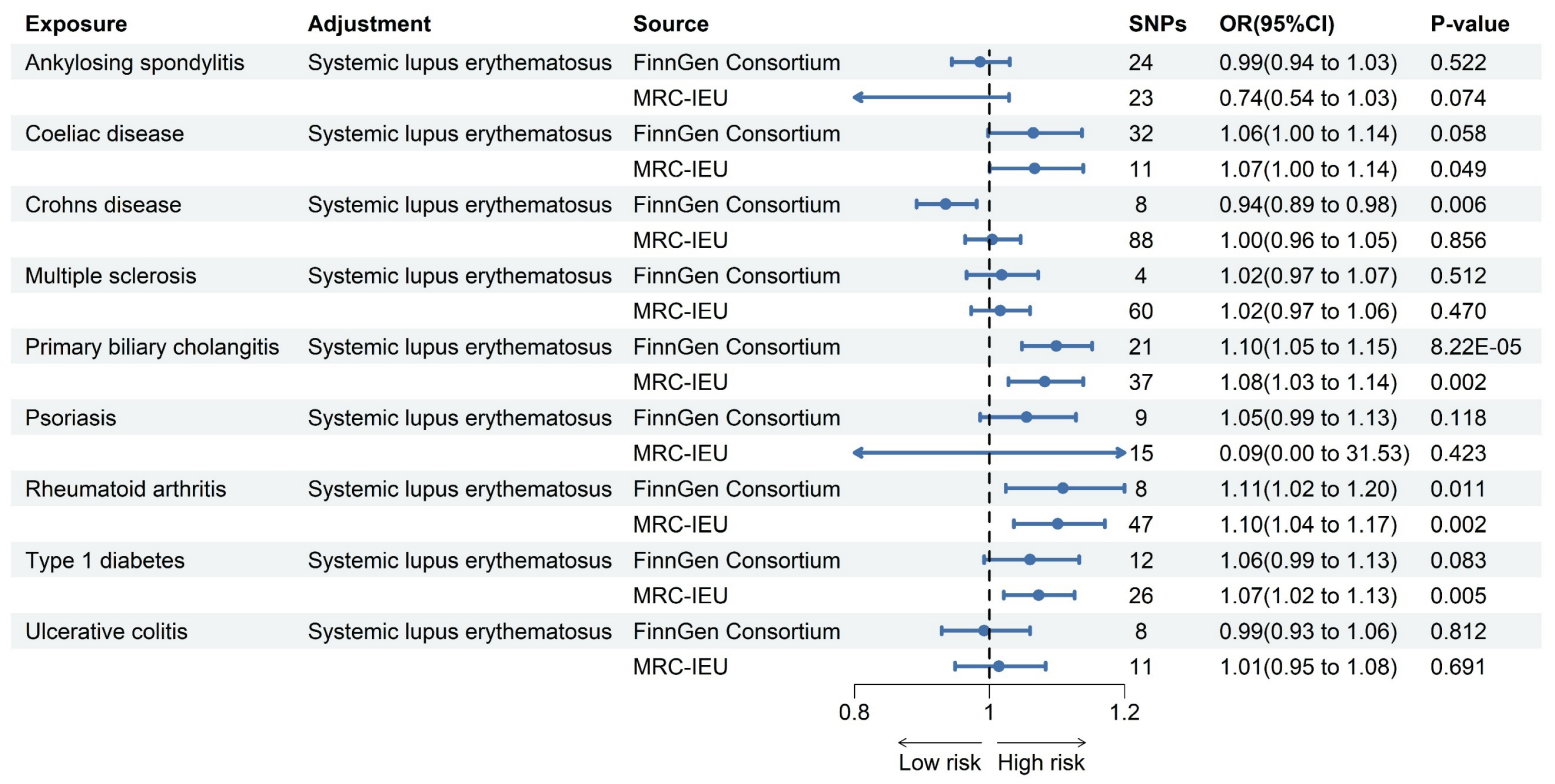
** Forest plot for MR analysis of PAH and ADs.** Forest plot to visualize the associations of genetically predicted autoimmune diseases with PAH using multivariable MR analyses. CI: 95% confidence interval. OR, odds ratio.

We performed a MVMR analysis of fourteen other ADs by individually adjusting for SLE. And primarily utilizing multivariable IVW to investigate the independent causal relationship between other ADs and PAH. As shown in the **Figure 2** after comparing the results obtained from two different pooled data analyzed using the multivariable IVW method, we found that only the genetically predicted PBC (OR = 1.10, 95% CI 1.05-1.15, *P* = 8.22E-5; OR = 1.08, 95% CI 1.03-1.14, *P* =0.002) and RA (OR = 1.11, 95% CI 1.02-1.20, *P* = 0.011; OR = 1.10, 95% CI 1.04-1.17, *P* = 0.002) maintained a positive result in contrast to the univariate MR results. Based on these results, we conclude that PBC and RA increase the risk of developing PAH independently of the effects of other ADs.

#### Intermediary analysis

**Figure 3:**
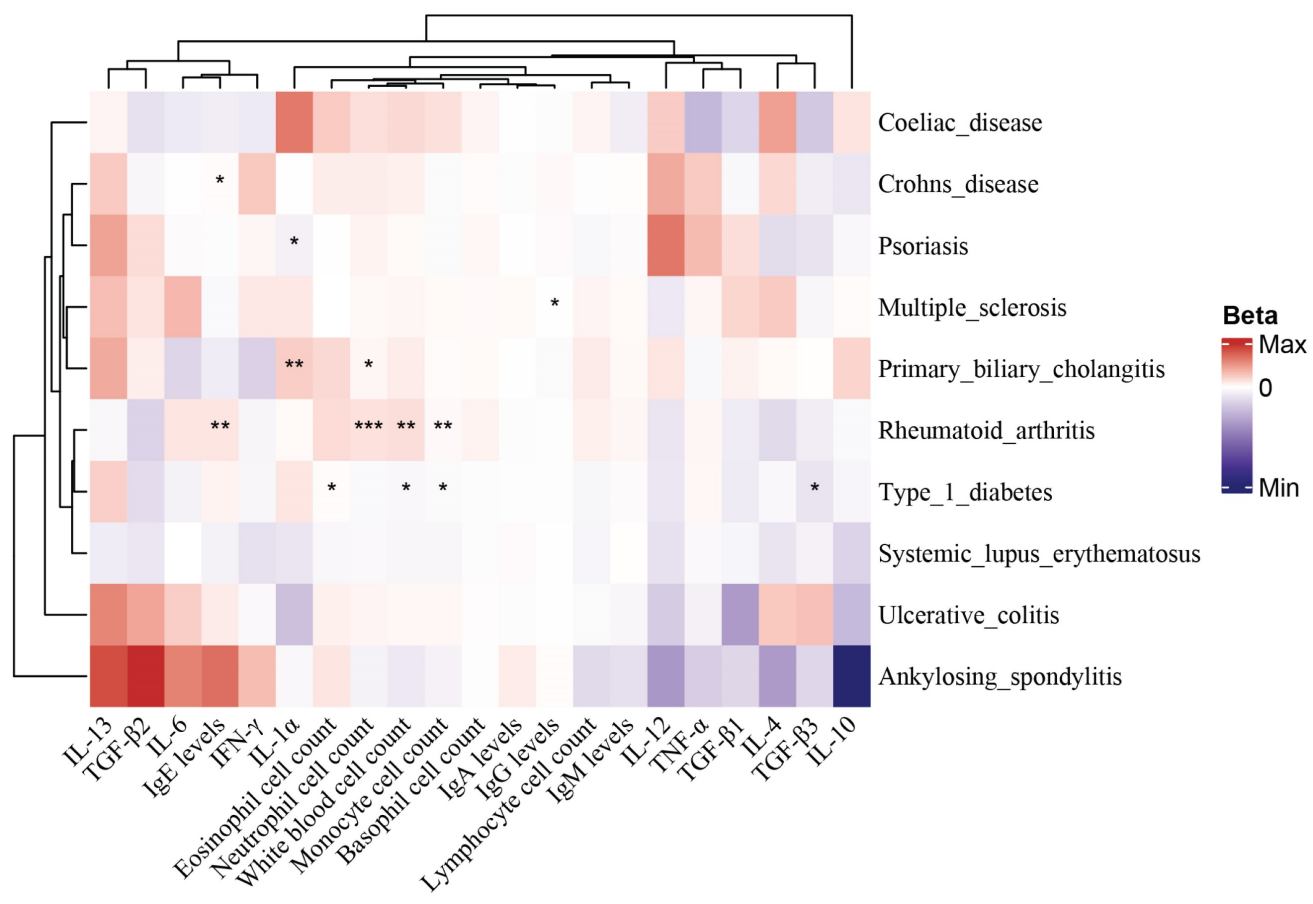
** Heat map of MR between mediator risk and ADs.** The heat map displays the MR analysis results of ADs and mediators. The color represents the β estimators of MR analysis, where blue represents a decreased mediator risk and red represents an increased mediator risk. ***: P < 0.001; **: P < 0.01; *: 0.05 < P < 0.01.

To determine whether ADs induce PAH through potential mediators, 21 potential mediators that may be altered by ADs were included in this study, including 6 blood cell counts, 4 immunoglobulins, and 11 inflammatory cytokines, as shown in **Table S2**. Two-step MR was used to explore the extent to which ADs affect PAH through 21 potential mediators, and the results of the analysis are displayed in **Table S8-Table S13** and **Figure 3**.

#### Reverse MR

Immune dysfunction is also a prevalent characteristic of PAH^34^. Researchers and scholars believe PAH has been associated with immune dysfunction^35^. According to a prior study^36^, immune dysfunction was found to result in a higher occurrence of certain ADs. In reverse MR analysis, the IVW method detected a significant causal relationship between genetically predicted PAH and SLE (OR = 1.090, 95% CI = 1.014-1.171, *P* = 0.014). In addition, we did not observe reverse causality between PAH and the other nine ADs **(Figure S3).**

### Observational study

**Table 1** presents the descriptive characteristics of the patients included in our study cohort, which totals 53,569 individuals diagnosed with ADs, of whom only 2,689 developed PH.

In this study, we employed a multivariate logistic regression model to adjust for potential confounding factors such as demographic data (age, gender, race), vital signs (heart rate, temperature, respiratory rate, blood oxygen saturation), comorbidities (hypertension, diabetes), and severity of disease scores (OASIS, SOFA), aiming to refine the accuracy of our risk predictions for PH. The adjusted results presented in **Table 2**, where our analysis revealed that SLE (OR = 2.19, 95% CI 1.52-3.15, *P* < 0.001), RA (OR = 1.39, 95% CI 1.11-1.75, *P* = 0.005), CD (OR = 2.22, 95% CI 1.42-3.48, *P* = 0.001), psoriasis (OR = 1.79, 95% CI 1.37-2.33, *P* < 0.001), and T1DM (OR = 1.84, 95% CI 1.34-2.52, *P* < 0.001) were significantly associated with a higher risk of developing PH.

### Estimation of the genetic correlation of pulmonary hypertension with T1DM and PBC

**Figure 4:**
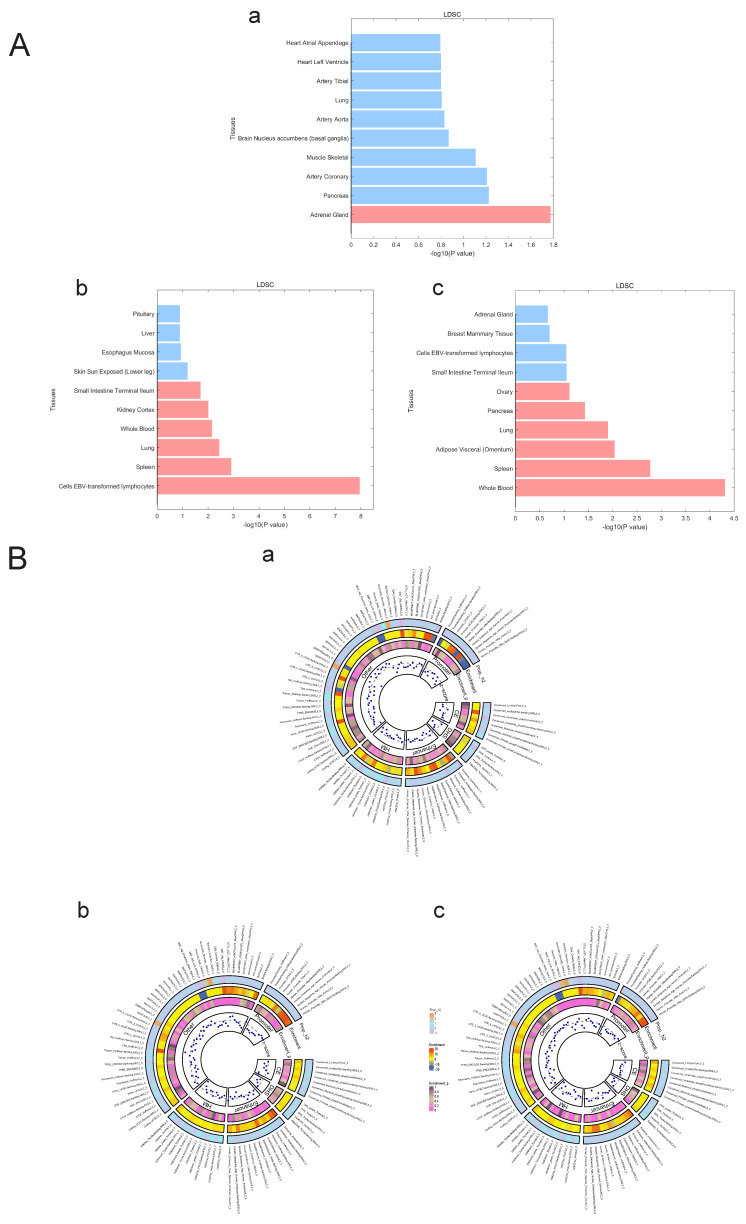
** A. LDSC tissue type specificity analysis regarding PAH, PBC and T1DM.** The analysis results of LDSC tissue type specificity analysis are represented by horizontal bar charts, with red representing P<0.05 and blue representing P>0.05. a: PAH, b: PBC, c: T1DM. **B. LDSC partitioned heritability analysis regarding PAH, PBC and T1DM.** The analysis results of different genetic regions of ADs and PAH are represented by a circular heatmap. Colors represent numerical values. a: PAH, b: PBC, c: T1DM.

**Figure 5:**
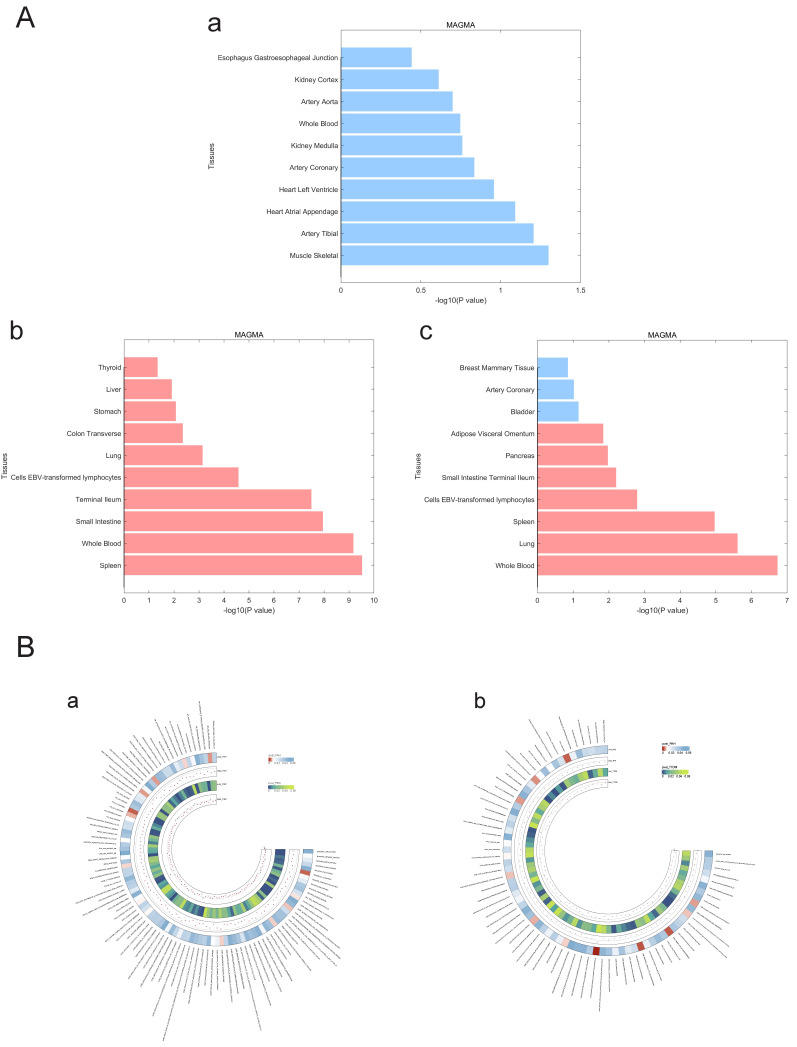
** A. MAGMA tissue type specificity analysis regarding PAH, PBC and T1DM.** The analysis results of Magma tissue type specificity analysis are represented by horizontal bar charts, with red representing P < 0.05 and blue representing P > 0.05. a: PAH, b: PBC, c: T1DM. **B. MAGMA pathway analysis between PAH and ADs.** The circular heatmap shows positive results (P < 0.05) for both PAH and ADs in the analysis of the Magma pathway. The color of the heatmap represents the magnitude of the P-value. a: PAH & PBC, b: PAH & T1DM.

**Figure 6:**
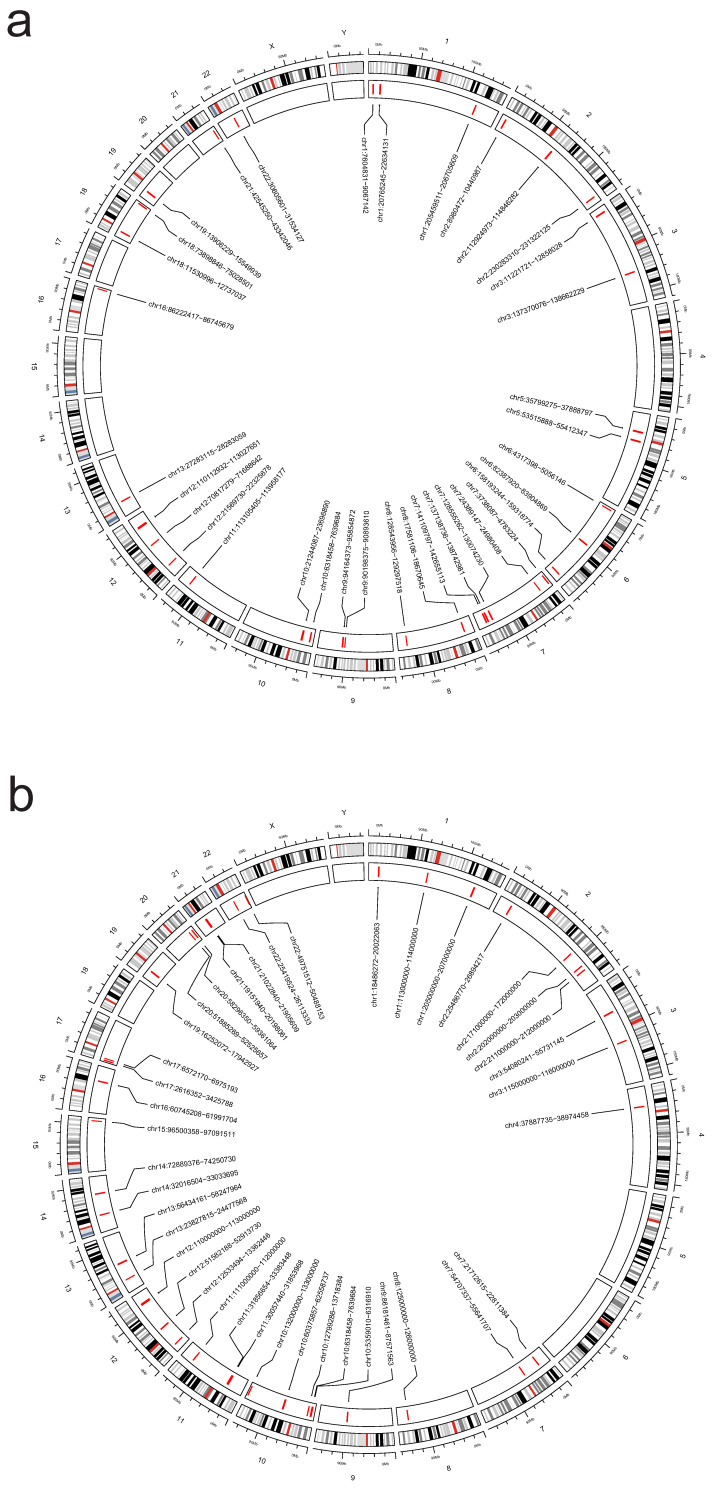
** SUPERGNOVA analysis between PAH and ADs.** The outer circle of the circular diagram represents chromosomal locations, while the inner red regions indicate chromosomal segments where there is inter-disease genetic linkage between autoimmune diseases (ADs) and pulmonary arterial hypertension (PAH) in the Supergnova analysis. a: PAH & T1DM, b: PAH & PBC.

We used LDSC to estimate the genetic correlation (unconstrained intercept**, Table 3**) between PAH and T1DM, PBC (T1DM: rg=0.32 *P*=1.20x10^-8^; PBC: rg=0.26, *P*=1.20x10^-8^ LDSC.) where LDSC tissue type specific is shown in **Figure 4A** and LDSC partitioned heritability tissue type specific is shown in **Figure 4B** and** Table 3**. In addition to this, the method of MAGMA tissue type specific analysis and pathway are equally analyzed with significance in **Figure 5A and Figure 5B**. Analyses by SUPERGNOVA and Genetic covariance analyzer (GNOVA) also demonstrated a positive and consistent genetic association. See **Figure 6** and **Table 4** for details.

### Identification of genomic regions

**Figure 7:**
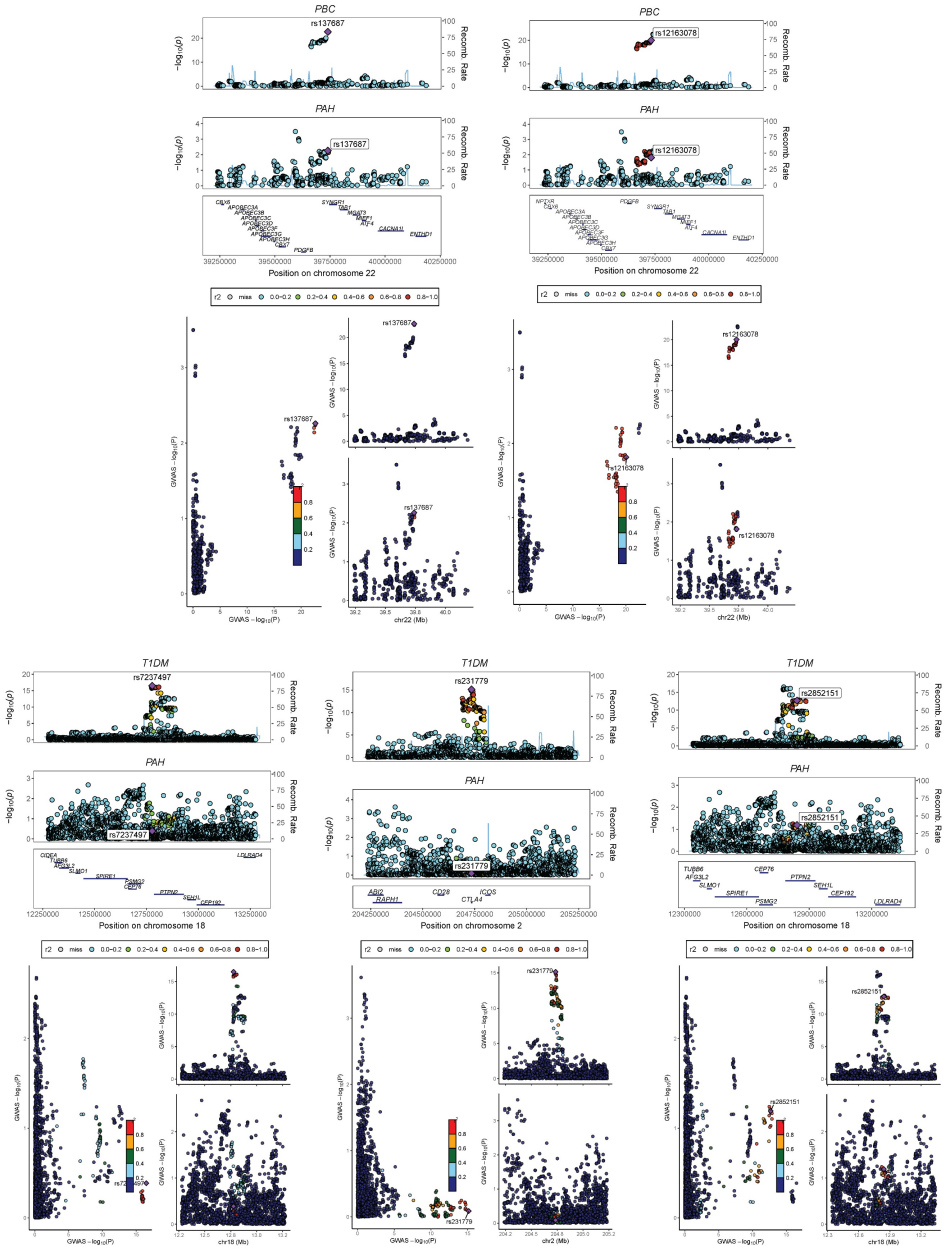
**Bayesian colocalization analysis between PAH and Ads.** Perform Bayesian colocalization analysis on the results from CPASSOC and MATG analyses. PAH&T1DM:rs2046862; PAH&PBC:rs137687 and rs12163078.

We performed a multi-trait analysis of GWAS (MTAG) to enhance our ability to identify genetic SNPs shared across traits. For PAH and PBC, both MTAG and cross-phenotype association tests (CPASSOC) revealed a total of 1810 genome-wide significant SNPs (P < 5 × 10-8). For PAH and T1DM, both methods identified 13148 genome-wide significant SNPs (P < 5 × 10-8), including 5 newly discovered shared SNPs (T1DM: rs231779, rs2852151, rs7237497; PBC: rs137687, rs12163078). The maximum FDR values for MTAG analyses were 4.98E^-8^ and 9.60E^-11^ for PAH and PBC, respectively; and 4.99E-8 and 1.07E-8 for PAH and T1DM, respectively. Furthermore, the MTAG results closely aligned with those obtained from CPASSOC, indicating the reliability of MTAG findings and minimal bias in its assumptions. In addition, 3 risk SNPs were found to be consistently significant when analyzed by ρ-HESS, with shared loci confirmed by Colocalization analysis (PPH4 > 0.4), as shown in **Figure 7** and **Table S1**.

**Figure 8:**
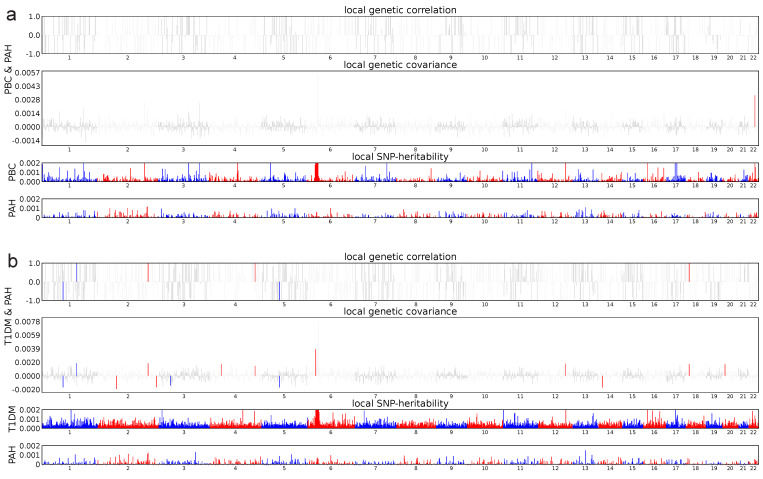
**ρ-HESS analysis between PAH and Ads.** Local genetic correlation between PAH and ADs. Manhattan plot showed the estimates of local genetic correlation and local genetic covariance between PAH and ADs, and, local SNP heritability of PAH and ADs, respectively. Red and blue bars in 'local genetic correlation' and 'local genetic covariance' represent significant regions which shared SNP heritability, after multiple adjustment (P < 5E-08 in both local SNP heritability test, and, P < (0.05/ the number of regions) in local genetic covariance test). a: PAH & PBC, b: PAH & T1DM.

In terms of estimating local genetic correlations, 15 significant regions were identified in PAH and T1DM (*P* < 0.05, ρ-HESS), and 1 significant region was found in PAH and PBC (*P* < 0.05, ρ-HESS). The genome-wide local genetic correlations computed by ρ-HESS were largely consistent with those obtained using LDSC, as detailed in **Figure 8**.

### Identification of shared functional genes

**Figure 9:**
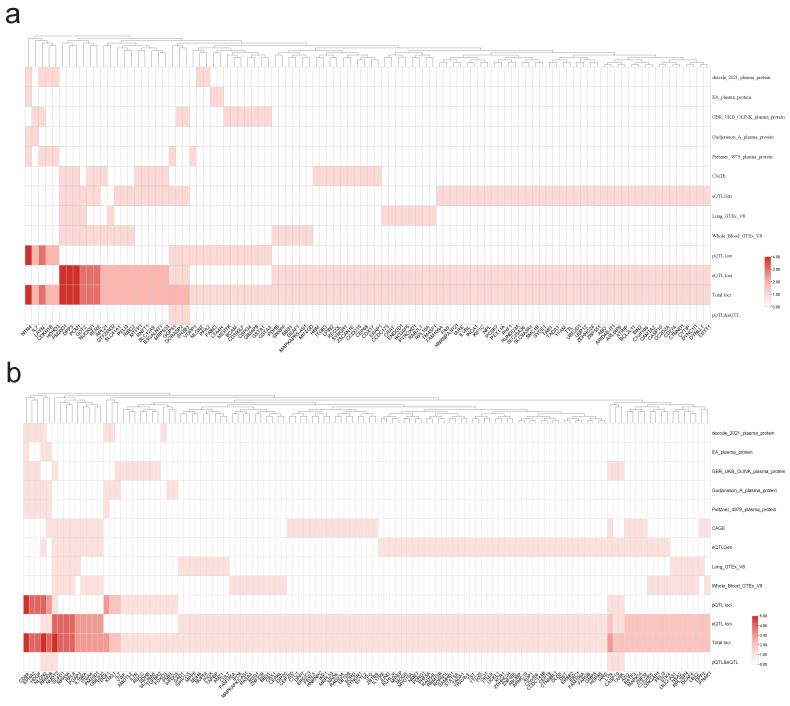
** SMR analysis.** To be selected as candidate (double positive in ADs and PAH) genes for Heat Map, both autoimmune diseases and pulmonary arterial hypertension must satisfy the following criteria: P_(SMR)_ < 0.05, P_(HEDI)_ > 0.05. a: PAH & PBC, b: PAH & T1DM.

**Figure 10:**
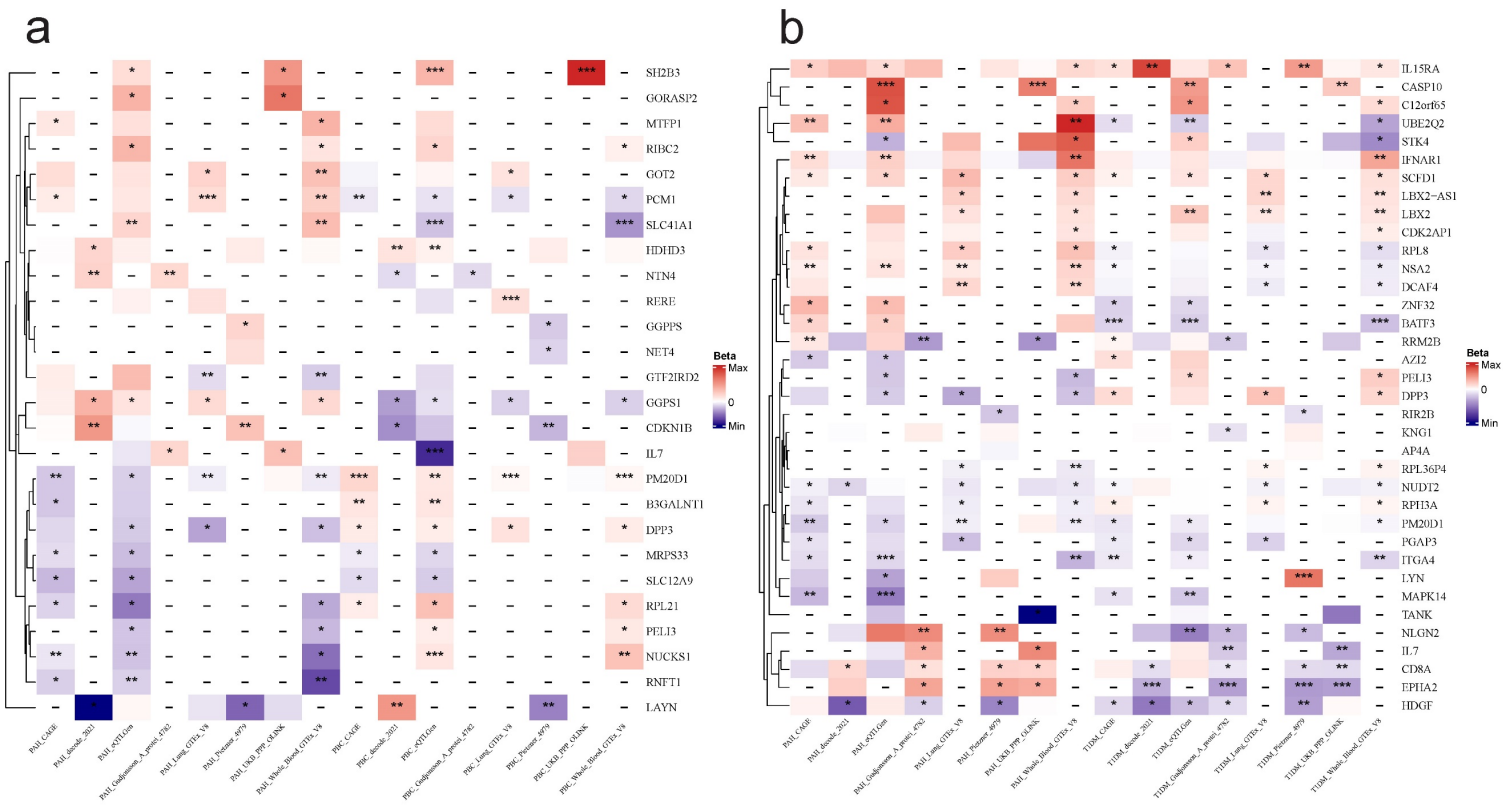
** Cis-MR analysis between candidate genes and diseases.** The heat map displays the cis-MR analysis results of candidate genes and diseases. The color represents the β estimators of cis-MR analysis. ***: P < 0.001; **: P < 0.01; *: 0.05 < P < 0.01. “-”: This tissue or data source lacks data for this gene. a: PAH & PBC, b: PAH & T1DM.

By conducting a joint analysis of GWAS merged data from eQTLGen and GTEx, along with merged whole-blood eQTL data, we applied SMR to infer the causal relationships between PAH and T1DM, as well as PBC. We identified a total of 45 genes that exhibited dual positivity (values > 2) in both PAH and PBC, with NTN4, PM20D1, and DPP3 showing strong positive signals. In the case of PAH and T1DM, we found 68 genes meeting the criteria for dual positivity, with strong positives including CD8A, HDGF, and EPHA2, as detailed in **Figure 9**. Under the conditions for SMR analysis, we conducted cis-MR analysis on the dual-positive genes to further infer the relationships between PAH and T1DM, as well as PBC.** Figure 10** shows that there are 21 genes exhibiting dual positivity between PAH and PBC, with PM20D1 and NUCKS1 being the most significant. Additionally, we identified 33 dual-positive genes between PAH and T1DM, with CASP10 and BATF3 serving as representative examples.

**Figure 11:**
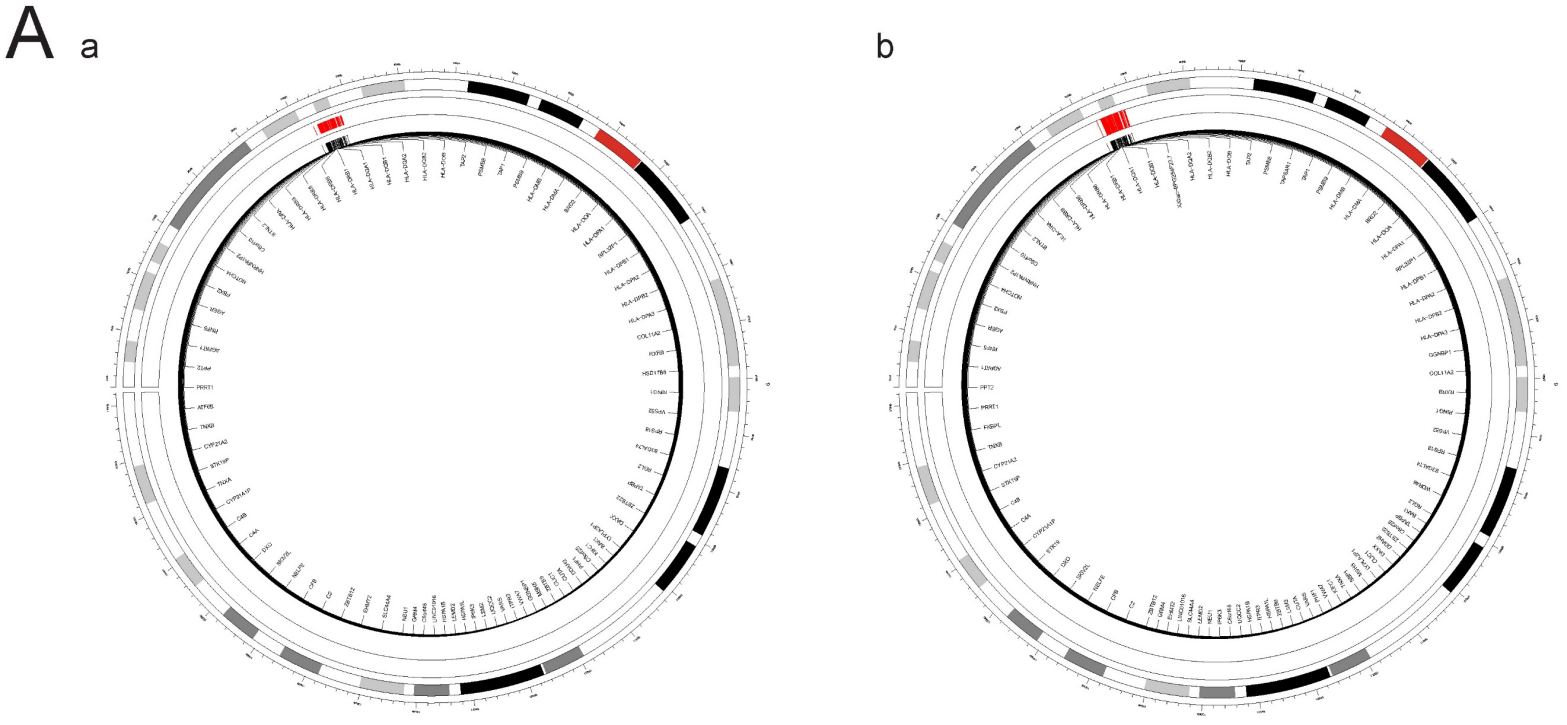

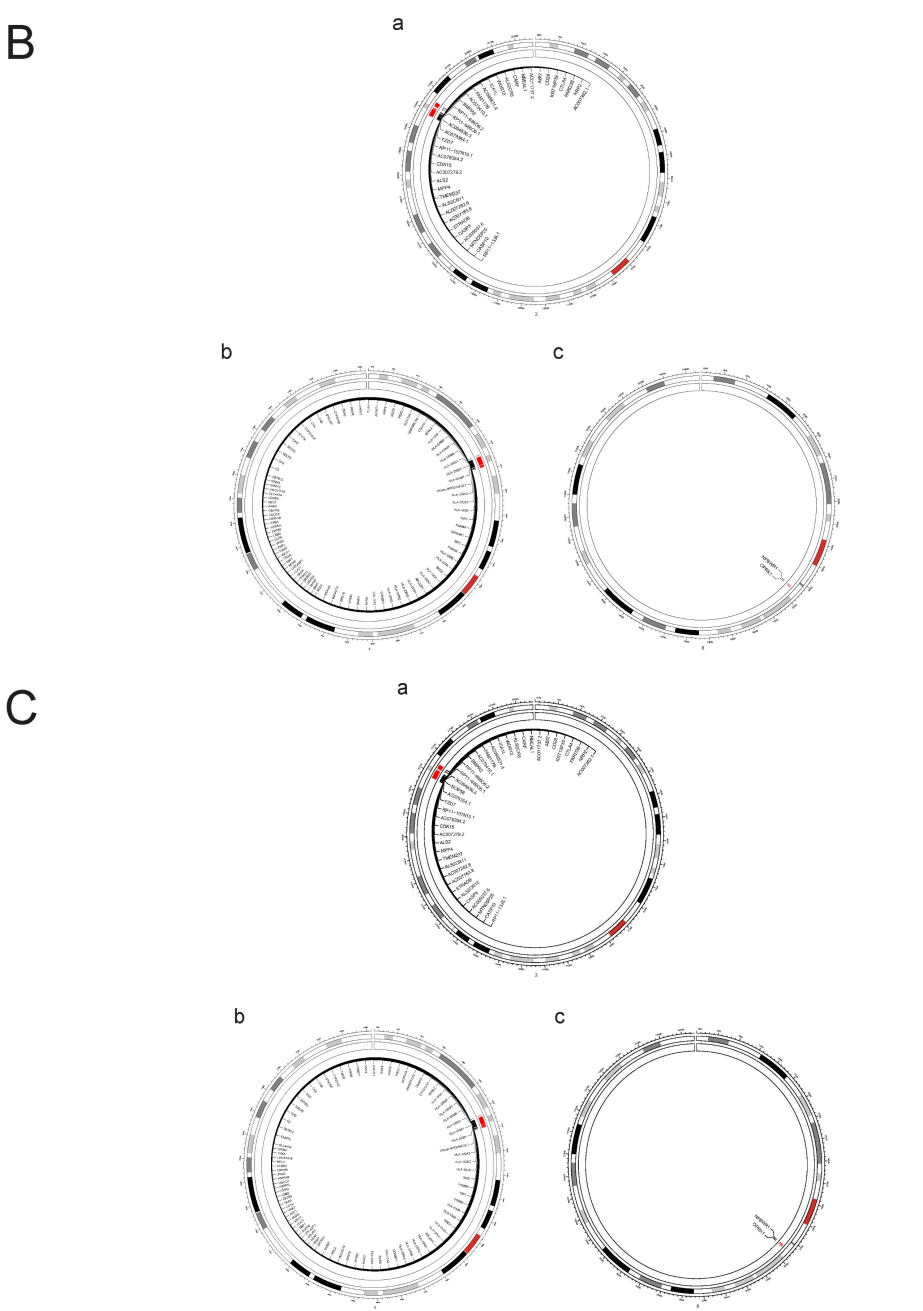
** A. FOCUS analysis of relevant lung tissue and whole blood between PAH and PBC.** The outer circle of the circular diagram represents chromosomal locations, while the inner red regions indicate Fine mapping of causal gene sets (Focus) analysis for both PBC and PAH showed positive chromosomal positions (FDR_(PAH&PBC)_ > 0.05). **B. FOCUS analysis of relevant lung tissue between PAH and T1DM.** The outer circle of the circular diagram represents chromosomal locations, while the inner red regions indicate Fine mapping of causal gene sets (Focus) analysis for both T1DM (Lung) and PAH showed positive chromosomal positions (FDR_(PAH&PBC)_ > 0.05). **C. FOCUS analysis of relevant whole blood between PAH and T1DM.** The outer circle of the circular diagram represents chromosomal locations, while the inner red regions indicate Fine mapping of causal gene sets (Focus) analysis for both T1DM (Whole blood) and PAH showed positive chromosomal positions (FDR_(PAH&PBC)_ > 0.05).

Through FOCUS analysis of pathogenic gene sets, we identified 88 genotypes that exhibit dual positivity for PAH and PBC, as shown in **Figure 11A**. For PAH and T1DM, we found 130 dual-positive genotypes, illustrated in **Figure 11B**. Additionally, there are 130 dual-positive genotypes for PAH and T1DM (whole blood), presented in **Figure 11C**. These findings facilitate a more accurate understanding of the relationships between these diseases.

**Figure 12:**
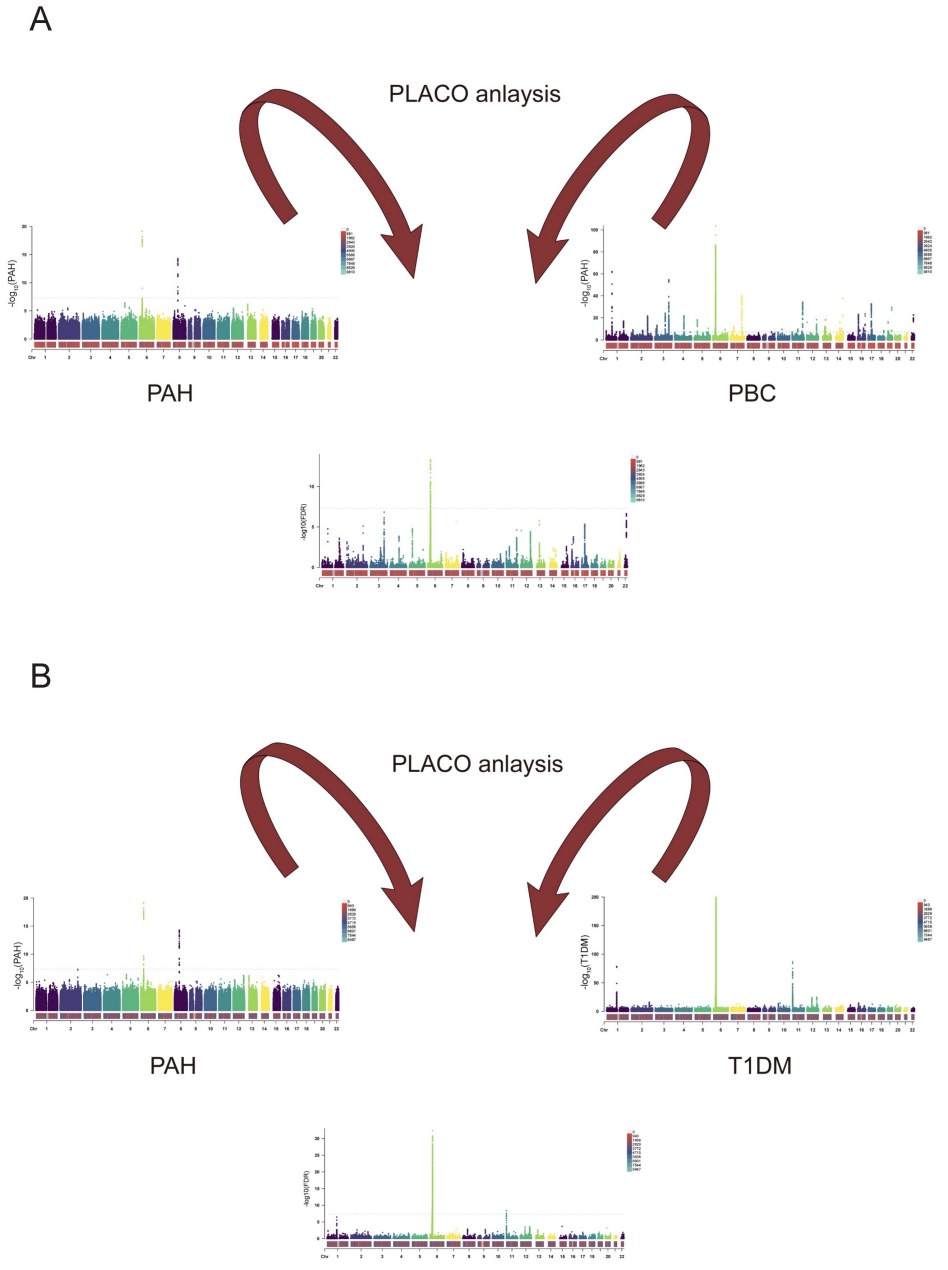
** A. PLACO analysis graphs for PAH and PBC.** The results of the placo analysis are represented by the Manhattan plot in the middle, and the bottom colors represent the number of SNPs at each chromosome position. Dashed lines indicate P-values or FDR values = 5E-8. **B. PLACO analysis graphs for PAH and T1DM.** The results of the placo analysis are represented by the Manhattan plot in the middle, and the bottom colors represent the number of SNPs at each chromosome position. Dashed lines indicate P-values or FDR values = 5E-8.

Finally, we conducted PLACO analysis to identify SNPs meeting FDR values < 5 × 10⁻⁸. Among these, 130 SNPs were found to be significant in the context of PAH and PBC, as illustrated in **Figure 12A**, while 888 SNPs were identified in relation to PAH and T1DM, as shown in **Figure 12B**. These data support our hypotheses regarding the relationships between these two diseases.

## Discussion

In this study, we present compelling evidence of causality and shared genetic architecture between PAH and ADs. Our findings offer novel insights into their co-occurrence, potentially advancing disease prediction, diagnosis, and treatment strategies.

We conducted a thorough assessment of the relationship between fifteen ADs and PH using a two-sample MR approach and real-world observational analysis. Our findings indicate a significant genetic causality linking PBC and T1DM with PH, suggesting that ADs may elevate the risk of PH. Mediation analyses revealed that none of the tested mediators could explain the effect of ADs on PH. Additionally, our extended reverse MR analysis identified genetic causality linking PH with SLE, PBC, and T1DM, suggesting that PH may be a risk factor for the development of these three ADs. However, no significant positive or negative genetic causality was found between PH and other ADs.

A retrospective cohort study indicated that individuals with mild psoriasis did not demonstrate an association with PH compared to healthy controls, whereas those with severe psoriasis exhibited a markedly elevated risk of PH^37^. Previous studies have identified diabetes as a potential risk factor for PH onset^38^, ^39^. However, Lopez-Lopez JG *et al.*^40^ demonstrated through animal experiments that the observed alterations in rats with T1DM were insufficient to induce a sustained increase in pulmonary arterial stress. Mohammad-Reza Movahed *et al.* 39 demonstrated in their study a notably higher prevalence of pulmonary embolism and pulmonary hypertension among diabetic patients. Additionally, Ahmad I M Al-Shafei *et al.* ^41^conducted an experiment illustrating that sustained elevation of pulmonary pressures results in compensatory right ventricular hypertrophy. They also observed that an increase in the weight of the right ventricle, combined with the weight of the nasal septum, indirectly contributes to the onset of type 1 diabetes mellitus^41^. There remains controversy regarding the impact of T1DM on PH, necessitating further research to clarify their relationship. Several case reports have highlighted an increased likelihood of PAH development in individuals with AS^42^, ^43^. Prabu A reported that PH represents a significant respiratory concern among individuals with SLE, with a prevalence ranging from approximately 4% to 5%. Furthermore, the incidence of PH was notably higher in patients diagnosed with both PBC and SLE compared to those with SLE alone^44^. Trapp CM *et al.* discovered a close association between hyperthyroidism and PAH^45^. Appropriate treatment of hyperthyroidism can improve or alleviate PH^45^-^47^. Moreover, compared with PAH patients who have hypothyroidism, those without hypothyroidism tend to have a better prognosis, exhibiting enhanced functional capacity and improved pulmonary hemodynamics^14^.

Despite its valuable insights, this study has inherent limitations. The study focused narrowly on examining the causal relationship between common ADs and PH, omitting a comprehensive assessment of all ADs. However, to expand the scope of the analysis, we included additional diseases, such as Graves' disease, Hashimoto's thyroiditis, systemic sclerosis, mixed connective tissue disease, and Sjögren's syndrome, by relaxing the sample size threshold to 500 cases for these diseases. While this adjustment allowed us to include these diseases, the smaller sample sizes may introduce potential instability in the results, increasing the risk of false positives or false negatives. Furthermore, no real-world data were available to validate these findings, which limits our ability to confirm their reliability in clinical practice. Additionally, due to the scarcity of SNPs meeting stringent inclusion criteria (P < 5e-08, r² < 0.01, kb > 10,000) in certain ADs like Crohn's disease, multiple sclerosis, and SLE, we slightly relaxed the selection criteria to P < 5e-06. However, all included instrumental variables had F-statistics greater than 10, ensuring robustness against weak instrumental bias.

Furthermore, our analysis identified a significant causal link between primary biliary cholangitis (PBC) and PH. In a bidirectional multivariate mendelian experiment, Lin *et al.* observed that primary biliary cholangitis (PBC) may play a role in the development of type 2 diabetes (T2DM) and several cardiovascular diseases (CVDs), and it shows a genetic correlation with type 1 diabetes mellitus (T1DM)^48^.Nonetheless, the limitations of the MIMIC-IV database, particularly its lack of detailed PBC data, hindered validation through observational studies. Looking ahead, we advocate for future research efforts to prioritize comprehensive data collection on PBC patients to substantiate the impact of PBC on the development of PH.

## Conclusion

In conclusion, our study reveals a significant causal relationship and genetic correlation between pulmonary arterial hypertension (PAH) and autoimmune diseases (ADs). We identified shared risk SNPs among PAH, type 1 diabetes mellitus (T1DM), and primary biliary cholangitis (PBC). Additionally, we pinpointed genes that exhibit dual positivity in both PAH and PBC, as well as in PAH and T1DM. These findings provide insights into the shared genetic basis of PAH and AD, enhancing our understanding of their pathogenesis and interrelationships.

## Supplementary Material

Supplemental Methods & efigures.

Supplementary tables for cis-MR.

Supplementary tables for Fine mapping of causal gene sets.

Supplementary tables for Hess & colocalization.

Supplementary tables for MR1.

Supplementary tables for MR2.

Supplementary tables for SMR.

## Figures and Tables

**Table 1:** The baseline characteristics and demographic characteristics of the study population.

Variables	Total (n = 53569)	PH (n = 2689)	Non-PH (n = 50880)	*P*-value
Gender, n (%)				< 0.001
Female	23647 (44.1)	1377 (51.2)	22270 (43.8)	
Male	29922 (55.9)	1312 (48.8)	28610 (56.2)	
Race, n (%)				< 0.001
Asian	1580 (2.9)	68 (2.5)	1512 (3.0)	
Black	4902 (9.2)	434 (16.1)	4468 (8.8)	
White	36059 (67.3)	1756 (65.3)	34303 (67.4)	
Other	11028 (20.6)	431 (16.0)	10597 (20.8)	
Age, Median (IQR)	66.0 (54.0, 78.0)	73.0 (62.0, 82.0)	66.0 (54.0, 78.0)	< 0.001
Vital signs				
Temperature (°C), Median (IQR)	36.7 (36.4, 37.1)	36.7 (36.4, 36.9)	36.7 (36.4, 37.1)	0.007
HR, Mean (SD)	87.7 (20.0)	88.2 (20.2)	87.7 (19.9)	0.192
RR, Mean (SD)	18.9 (5.9)	20.0 (6.2)	18.9 (5.8)	< 0.001
SpO_2_ (%), Median (IQR)	98.0 (96.0, 100.0)	97.0 (94.0, 100.0)	98.0 (96.0, 100.0)	< 0.001
Severity of illness				
SOFA, Mean (SD)	4.3 (3.4)	5.0 (3.4)	4.2 (3.4)	< 0.001
OASIS, Median (IQR)	30.0 (24.0, 36.0)	30.0 (25.0, 36.0)	30.0 (24.0, 36.0)	< 0.001
Comorbidities, n (%)				
Hypertension				< 0.001
No	20155 (37.6)	613 (22.8)	19542 (38.4)	
Yes	33399 (62.4)	2076 (77.2)	31323 (61.6)	
Diabetes				< 0.001
No	39601 (73.9)	1704 (63.4)	37897 (74.5)	
Yes	13953 (26.1)	985 (36.6)	12968 (25.5)	

^a^Data are reported as the mean ± SD or median (interquartile range) or N (%). ^b^Abbreviation: HR: Heart rate; RR: Respiratory rate; SpO_2_: Oxygen saturation; SOFA: sequential organ failure assessment; OASIS: Oxford Acute Severity of Illness Score. ^c^*P* - value less than 0.05 is expressed in bold.

**Table 2:** The relationship between autoimmune diseases and the occurrence of pulmonary hypertension.

Autoimmune diseases	OR (95%CI)	P-value
Systemic lupus erythematosus	2.19 (1.52-3.15)	<0.001
Rheumatoid arthritis	1.39 (1.11-1.75)	0.005
Crohn's disease	2.22 (1.42-3.48)	0.001
Psoriasis	1.79 (1.37-2.33)	<0.001
Type 1 diabetes	1.84 (1.34-2.52)	<0.001
Ankylosing spondylitis	1.60 (0.69-3.71)	0.274
Celia disease	1.62 (0.93-2.82)	0.088
Multiple sclerosis	0.68 (0.35-1.32)	0.251
Ulcerative colitis	0.59 (0.32-1.07)	0.084

^a^Abbreviation: OR: odds ratio; CI: confidence interval. *^b^P* - value less than 0.05 is expressed in bold.

**Table 3:** Analysis of LDSC (test without constrained intercept) on pulmonary hypertension and both PBC and T1DM

LDSC test without constrained intercept	
Heritability of PBC	
Total Observed scale h2	0.3569 (0.0485)
Lambda GC	1.0315
Mean Chi^2	1.1978
Intercept	constrained to 1
Heritability of phenotype 2/2	
Total Observed scale h2	0.28 (0.0317)
Lambda GC	1.071
Mean Chi^2	1.068
Intercept	constrained to 1
Genetic Covariance	
Total Observed scale gencov	0.081 (0.0167)
Mean z1*z2	0.0283
Intercept	constrained to 0
Genetic Correlation	
Genetic Correlation	0.2563 (0.0523)
Z-score	4.9037
P	9.4046e-07
LDSC test without constrained intercept	
Heritability of T1DM	
Total Observed scale h2	0.559 (0.0439)
Lambda GC	1.127
Mean Chi^2	1.1946
Intercept	constrained to 1
Heritability of phenotype 2/2	
Total Observed scale h2	0.2965 (0.0286)
Lambda GC	1.0649
Mean Chi^2	1.0648
Intercept	constrained to 1
Genetic Covariance	
Total Observed scale gencov	0.1285 (0.0188)
Mean z1*z2	0.034
Intercept	constrained to 0
Genetic Correlation	
Genetic Correlation	0.3155 (0.0465)
Z-score	6.7805
P	1.1978e-11

P: P-value.

**Table 4:** Regarding the Gnova analysis of pulmonary hypertension and both PBC and T1DM

GNOVA			
PAH&PBC		T1DM&PBC	
rho	0.05674622	rho	0.09952875
se_rho	0.01401888	se_rho	0.01802472
pvalue	5.169205e-05	pvalue	3.355615e-08
corr	0.2239634	corr	0.2643645
h2_1	0.2126004	h2_1	0.2960249
h2_2	0.3019644	h2_2	0.4788092
p	511841	p	1072518
P0	511841	P0	1072518

## References

[1] Hassoun PM (2021). Pulmonary arterial hypertension. The New England journal of medicine.

[2] Hoeper MM, Ghofrani HA, Grünig E, Klose H, Olschewski H, Rosenkranz S (2017). Pulmonary hypertension. Deutsches Arzteblatt international.

[3] Humbert M, Sitbon O, Simonneau G (2004). Treatment of pulmonary arterial hypertension. The New England journal of medicine.

[4] Swinnen K, Quarck R, Godinas L, Belge C, Delcroix M (2019). Learning from registries in pulmonary arterial hypertension: Pitfalls and recommendations. European respiratory review: an official journal of the European Respiratory Society.

[5] Li L, Jick S, Breitenstein S, Hernandez G, Michel A, Vizcaya D (2017). Pulmonary arterial hypertension in the USA: An epidemiological study in a large insured pediatric population. Pulmonary circulation.

[6] Mercurio V, Bianco A, Campi G, Cuomo A, Diab N, Mancini A (2019). New drugs, therapeutic strategies, and future direction for the treatment of pulmonary arterial hypertension. Current medicinal chemistry.

[7] Galiè N, Channick RN, Frantz RP, Grünig E, Jing ZC, Moiseeva O (2019). Risk stratification and medical therapy of pulmonary arterial hypertension. The European respiratory journal.

[8] Marrack P, Kappler J, Kotzin BL (2001). Autoimmune disease: Why and where it occurs. Nature medicine.

[9] Rose NR (2016). Prediction and prevention of autoimmune disease in the 21st century: A review and preview. American journal of epidemiology.

[10] Yao M, Zhang C, Gao C, Wang Q, Dai M, Yue R (2021). Exploration of the shared gene signatures and molecular mechanisms between systemic lupus erythematosus and pulmonary arterial hypertension: Evidence from transcriptome data. Frontiers in immunology.

[11] Naranjo M, Mercurio V, Hassan H, Alturaif N, Cuomo A, Attanasio U (2022). Causes and outcomes of icu hospitalisations in patients with pulmonary arterial hypertension. ERJ open research.

[12] Sztrymf B, Souza R, Bertoletti L, Jaïs X, Sitbon O, Price LC (2010). Prognostic factors of acute heart failure in patients with pulmonary arterial hypertension. The European respiratory journal.

[13] Batton KA, Austin CO, Bruno KA, Burger CD, Shapiro BP, Fairweather D (2018). Sex differences in pulmonary arterial hypertension: Role of infection and autoimmunity in the pathogenesis of disease. Biology of sex differences.

[14] Vrigkou E, Vassilatou E, Dima E, Langleben D, Kotanidou A, Tzanela M (2022). The role of thyroid disorders, obesity, diabetes mellitus and estrogen exposure as potential modifiers for pulmonary hypertension. Journal of clinical medicine.

[15] Krieg VJ, Hobohm L, Liebetrau C, Guth S, Kölmel S, Troidl C (2020). Risk factors for chronic thromboembolic pulmonary hypertension - importance of thyroid disease and function. Thrombosis research.

[16] Bonderman D, Wilkens H, Wakounig S, Schäfers HJ, Jansa P, Lindner J (2009). Risk factors for chronic thromboembolic pulmonary hypertension. The European respiratory journal.

[17] Chu JW, Kao PN, Faul JL, Doyle RL (2002). High prevalence of autoimmune thyroid disease in pulmonary arterial hypertension. Chest.

[18] Lecka-Ambroziak A, Kot K (2024). Pulmonary hypertension and hypothyroidism-still an important clinical coincidence in paediatric population, an endocrinologist's point of view. Life (Basel, Switzerland).

[19] Wang RR, Yuan TY, Wang JM, Chen YC, Zhao JL, Li MT (2022). Immunity and inflammation in pulmonary arterial hypertension: From pathophysiology mechanisms to treatment perspective. Pharmacological research.

[20] Hu Y, Chi L, Kuebler WM, Goldenberg NM (2020). Perivascular inflammation in pulmonary arterial hypertension. Cells.

[21] Frid MG, Thurman JM, Hansen KC, Maron BA, Stenmark KR (2020). Inflammation, immunity, and vascular remodeling in pulmonary hypertension; evidence for complement involvement?. Global cardiology science & practice.

[22] Grimes DA, Schulz KF (2002). Cohort studies: Marching towards outcomes. Lancet (London, England).

[23] Irony TZ (2018). Case-control studies: Using "real-world" evidence to assess association. Jama.

[24] Emdin CA, Khera AV, Kathiresan S (2017). Mendelian randomization. Jama.

[25] Skrivankova VW, Richmond RC, Woolf BAR, Davies NM, Swanson SA, VanderWeele TJ (2021). Strengthening the reporting of observational studies in epidemiology using mendelian randomisation (strobe-mr): Explanation and elaboration. BMJ (Clinical research ed.).

[26] Johnson AEW, Bulgarelli L, Shen L, Gayles A, Shammout A, Horng S (2023). Mimic-iv, a freely accessible electronic health record dataset. Scientific data.

[27] Gazal S, Marquez-Luna C, Finucane HK, Price AL (2019). Reconciling s-ldsc and ldak functional enrichment estimates. Nature genetics.

[28] Bulik-Sullivan B, Finucane HK, Anttila V, Gusev A, Day FR, Loh PR (2015). An atlas of genetic correlations across human diseases and traits. Nature genetics.

[29] de Leeuw CA, Mooij JM, Heskes T, Posthuma D (2015). Magma: Generalized gene-set analysis of gwas data. PLoS computational biology.

[30] Shi H, Mancuso N, Spendlove S, Pasaniuc B (2017). Local genetic correlation gives insights into the shared genetic architecture of complex traits. American journal of human genetics.

[31] Ray D, Chatterjee N (2020). A powerful method for pleiotropic analysis under composite null hypothesis identifies novel shared loci between type 2 diabetes and prostate cancer. PLoS genetics.

[32] Krishnamoorthy S, Li GH, Cheung CL (2023). Transcriptome-wide summary data-based mendelian randomization analysis reveals 38 novel genes associated with severe covid-19. Journal of medical virology.

[33] Skene NG, Grant SG (2016). Identification of vulnerable cell types in major brain disorders using single cell transcriptomes and expression weighted cell type enrichment. Frontiers in neuroscience.

[34] Chen CN, Hajji N, Yeh FC, Rahman S, Ali S, Wharton J (2023). Restoration of foxp3(+) regulatory t cells by hdac-dependent epigenetic modulation plays a pivotal role in resolving pulmonary arterial hypertension pathology. American journal of respiratory and critical care medicine.

[35] Ormiston ML, Chang C, Long LL, Soon E, Jones D, Machado R (2012). Impaired natural killer cell phenotype and function in idiopathic and heritable pulmonary arterial hypertension. Circulation.

[36] Aspinall R, Andrew D (2000). Immunosenescence: Potential causes and strategies for reversal. Biochemical Society transactions.

[37] Choi YM, Famenini S, Wu JJ (2017). Incidence of pulmonary arterial hypertension in patients with psoriasis: A retrospective cohort study. The Permanente journal.

[38] Makarevich AE, Valevich VE, Pochtavtsev AU (2007). Evaluation of pulmonary hypertension in copd patients with diabetes. Advances in medical sciences.

[39] Movahed MR, Hashemzadeh M, Jamal MM (2005). The prevalence of pulmonary embolism and pulmonary hypertension in patients with type ii diabetes mellitus. Chest.

[40] Lopez-Lopez JG, Moral-Sanz J, Frazziano G, Gomez-Villalobos MJ, Moreno L, Menendez C (2011). Type 1 diabetes-induced hyper-responsiveness to 5-hydroxytryptamine in rat pulmonary arteries via oxidative stress and induction of cyclooxygenase-2. The Journal of pharmacology and experimental therapeutics.

[41] Al-Shafei AI, Wise RG, Gresham GA, Bronns G, Carpenter TA, Hall LD (2002). Non-invasive magnetic resonance imaging assessment of myocardial changes and the effects of angiotensin-converting enzyme inhibition in diabetic rats. The Journal of physiology.

[42] Yang TY, Chen YH, Siao WZ, Jong GP (2022). Case report: A rare manifestation of pulmonary arterial hypertension in ankylosing spondylitis. Journal of personalized medicine.

[43] Hung YM, Cheng CC, Wann SR, Lin SL (2015). Ankylosing spondylitis associated with pulmonary arterial hypertension. Internal medicine (Tokyo, Japan).

[44] Cheng C, Wang Z, Wang L, Zhao J, Wang Q, Tian X (2021). Clinical characteristics and prognosis of concomitant systemic lupus erythematosus and primary biliary cholangitis. Clinical rheumatology.

[45] Trapp CM, Elder RW, Gerken AT, Sopher AB, Lerner S, Aranoff GS (2012). Pediatric pulmonary arterial hypertension and hyperthyroidism: A potentially fatal combination. The Journal of clinical endocrinology and metabolism.

[46] Mercé J, Ferrás S, Oltra C, Sanz E, Vendrell J, Simón I (2005). Cardiovascular abnormalities in hyperthyroidism: A prospective doppler echocardiographic study. The American journal of medicine.

[47] Marvisi M, Zambrelli P, Brianti M, Civardi G, Lampugnani R, Delsignore R (2006). Pulmonary hypertension is frequent in hyperthyroidism and normalizes after therapy. European journal of internal medicine.

[48] Lin YL, Yao T, Wang YW, Yu JS, Zhen C, Lin JF (2024). Association between primary biliary cholangitis with diabetes and cardiovascular diseases: A bidirectional multivariable mendelian randomization study. Clinics and research in hepatology and gastroenterology.

