# Supplementary material for: A study on the genetic comorbidity between autoimmune diseases and pulmonary hypertension: an observational study and POST-GWAS analysis
Source: Int J Med Sci. 2025 Feb 18;22(6):1344–62. doi: 10.7150/ijms.107884 (PMC11898843; doi:10.7150/ijms.107884)
Supplement: Supplementary file 1 — Supplemental Methods & efigures. [file ijmsv22p1344s1.pdf]

Supplemental Methods

2.1 Mendelian randomization

2.1.1 Study design

Figure 1 illustrates our study design and MR Hypothesis. The objective of this research endeavor was to examine the causal connection that exists between 10 autoimmune diseases (ADs) and PAH. MR was implemented in accordance with three hypotheses[1]. Assumption 1: Instrumental variables (IVs) are strongly correlated with exposure; Assumption 2: IVs are independent of potentially confounding variables; and Assumption 3: IVs only affect outcomes through exposure, not through any other means. In addition, we adhered to the recommendations of STROBE-MR [2] to guarantee the transparency and reproducibility of our study.

2.1.2 Data sources

ADs Ankylosing spondylitis, celiac disease, Crohn's disease (CD), multiple sclerosis (MS), primary biliary cholangitis (PBC), psoriasis, rheumatoid arthritis (RA), systemic lupus erythematosus (SLE), type 1 diabetes mellitus (T1DM), and ulcerative colitis (UC). We conducted a comparative analysis of the two pooled datasets, the former consisting of data obtained from the FinnGen Consortium (R9) (https://www.finngen.fi/fi) and the Gwas Catalog database (https://www.ebi.ac.uk/gwas/) and the latter from the MRC-IEU database (https://gwas.mrcieu.ac.uk/). Of these, the diagnosis of 8 autoimmune diseases in the Finngen Consortium was confirmed using the International Classification of Diseases codes (ICD-9, ICD-10) provided, and detailed information is summarised in **eTable 1**. The 10 autoimmune diseases included in the MRC-IEU database are available online with detailed information provided in the referenced papers[3-12].

Mediators To investigate the potential mechanisms underlying the genetic link between ADs and PAH, we further calculated 21 potential mediators for analysis, including blood cell counts, immunoglobulins, and inflammatory cytokines that may be affected by ADs. For blood cell counts, we acquired genome-wide association study (GWAS) data from the Blood Cell Consortium. Immunoglobulins' summary statistics were collected from a study conducted by Scepanovic P et al[13], involving 1,000 individuals of French metropolitan descent. Pooled GWAS data for inflammatory cytokines were obtained from the study by Suhre K et al. [14] for a total of 3080 individuals (including 1000 cases and 2080 controls). The specifics of all data sources can be found in **eTable 2**.

PAH GWAS summary data for PAH were retrieved from the GWAS Catalog (https://www.ebi.ac.uk/gwas/)[15] , comprising 11,744 individuals of European, with 2,085 having PAH and 9,659 serving as controls.

2.1.3 SNP selection

It is common knowledge that rigorous IVs selection is essential for MR analysis results to be reliable. To fulfil the three previously mentioned stringent assumptions, we conducted a series of quality control procedures to identify suitable single nucleotide polymorphisms (SNPs). First, we selected SNPs significantly associated with ADs ($P < 5\times10^{-8}$) as IVs in the MRC-IEU database. Second, we not only applied the thresholds of $r^2 < 0.01$ and Kb > 10,000 to eliminate linkage disequilibrium (LD)-linked genes, but we also computed the F-statistic of exposure, and IVs with F-statistics < 10 were excluded to avoid bias due to weaker genetic instrumentation. to prevent bias from weaker genetic instrumentation. The formula is as follows: $F = R^2(N-K-1)/K(1-R^2)$. Third, palindromic SNPs were excluded. Finally, SNPs that were not present in the resultant dataset or had inconsistent

alleles between exposure and outcome (e.g., T/C vs T/G) were excluded. The remaining selected SNPs were then used as instrumental variables for further analyses.

Nevertheless, when applying the threshold of $P < 5 \times 10^{-8}$ to screen for SNPs in pooled data from the FinnGen Consortium and Gwas Catalog, the SNPs linked to Crohn's disease, Multiple sclerosis, and Systemic lupus erythematosus were all below 3. Typically, MR studies necessitate a minimum of 4 SNPs as IVs[16]. Therefore, based on prior studys[17, 18], we applied a less stringent *P*-value criterion of $P < 5 \times 10^{-6}$ in the FinnGen Consortium and Gwas Catalogue databases to identify corresponding SNPs for Crohn's disease, Multiple sclerosis, and Systemic lupus erythematosus.

Similarly, reverse MR analysis revealed an insufficient number of SNPs when adhering to the strict inclusion criteria mentioned. Therefore, we implemented the following criteria: $P < 5 \times 10^{-6}$, $r^2 < 0.01$ in a window of clusters > 10,000 kb.

2.1.4 Other factors

To satisfy that IVs are independent of potential confounding variables[1], we performed a thorough search of the PhenoScanner database(www.phenoscanner.medschl.cam.ac.uk) for established associations between instrumental SNPs and potential confounding variables. Considerable genetic resemblance was identified among ADs and they are commonly linked to major histocompatibility complex (MHC) genes[19]. In the present study, our exposure factors were the 10 autoimmune diseases mentioned above, suggesting a strong association between alterations in MHC genes and ADs. Therefore, to mitigate the potential effects that are closely associated with autoimmune diseases, we need to exclude variants in the MHC region, that is the short arm of chromosome 6 that contains genes encoding molecules involved in antigen presentation[20]. However, this method may lead to horizontal pleiotropy and breach the three primary assumptions of Mendelian randomization. Thus, univariate MR analyses were performed on SNPs with excluded MHC loci to minimize the effect of confounding factors, and were utilized as the main univariate MR analysis results.

2.1.5 statistical analyses

In univariate MR analyses, five MR methods were employed to investigate the causal impact of ADs on PAH. In concrete terms, for each autoimmune disease, we calculated SNP-specific Wald ratios to estimate the effect of exposure on the outcome of each IV, defined as $\beta_{EXP\text{-}OUT} = \beta_{SNP\text{-}OUT}/\beta_{SNP\text{-}EXP}$. An inverse variance weighting (IVW) approach was utilized as the primary analysis to amalgamate the effect sizes for each IV. MR-Egger, Weighted median, Simple mode, and Weighted mode were used as supplementary methods or to see if their results were consistent with the direction of IVW[21]. In our sensitivity analyses, we performed MR-Egger regression to detect possible bias in directional pleiotropy. The intercept term of the MR-Egger regression shows the average pleiotropic effect across all genetic heritability variants ($p < 0.05$ indicated the presence of pleiotropy). Moreover, the MR-PRESSO method was utilized to assess and adjust for horizontal pleiotropy. To evaluate heterogeneity of effects, we analysed heterogeneity of MR Egger and IVW methods using Cochran's Q statistic, with $p > 0.05$ indicating no heterogeneity. Furthermore, we conducted leave-one-out analyses to verify whether individual SNPs significantly affected the estimation of causal effects by removing them individually.

We also conducted a multivariable Mendelian randomization (MVMR) analysis to determine the independent causal impact of ADs on PAH. This approach balanced the effects of similarly related or different risk factor categories on the results, thus producing more objective results. The primary

method used was IVW. Furthermore, we utilized two-step MR to determine the extent to which ADs influence PAH via potential mediators and the coefficient product method to compute mediating effects. First, the total effect of ADs on PAH risk was estimated using a one-way MR (coefficient β_{XY}), and estimate the effect of ADs on the potential medium (coefficient β_{XM}). X, M, and Y, respectively, denote exposure, mediator, and outcome. Second, the MVMR was used to estimate the direct effect of potential mediators on PAH (coefficient β_{MY}). The proportion of the mediated effect was calculated by dividing the indirect effect ($\beta_{XM}\times\beta_{MY}$) by the total effect ($\beta_{XY}$).

In addition, we extended the MR analysis to bi-directional causal inference between ADs and PAH by performing a reverse MR analysis with PAH as the exposure and each of the ten immune disorders as the outcome. The IVW method was assessed as the primary outcome. $P < 0.05$ was considered significant.

All statistical analyses were conducted utilizing Mendelian Randomization (0.4.2), TwoSampleMR (0.5.7), MRPRESSO (1.0), and MVMR (0.3) in R version 4.2.2.

2.2 Real-world observational analysis

2.2.1 Data sources

This study conducted a retrospective cohort analysis using data from the Medical Information Mart for Intensive Care-IV (MIMIC-IV) (version 1.0)[22], a publicly available database maintained jointly by Beth Israel Deaconess Medical Center and the Massachusetts Institute of Technology. The database encompasses de-identified medical records from more than 70,000 patients who were admitted to intensive care at the Beth Israel Deaconess Medical Center during the period from 2008 to 2019. Our authors fulfilled the criteria for database access and spearheaded the data extraction efforts. The study adheres to the Declaration of Helsinki, and the requirement for informed consent was waived due to anonymized data were analyzed. The Institutional Review Board of the Beth Israel Deaconess Medical Center approved this research.

2.2.2 Population

Our primary focus was on patients who developed PH during their initial hospital admission. We classified diagnoses for a total of 53,569 patients according to the International Classification of Diseases, Ninth Revision (ICD-9) and Tenth Revision (ICD-10), under codes 4160, I2720, I272, I2722, I2729, I2723, and I270. Employing PostgreSQL software (version 14.6), we meticulously extracted data from the MIMIC-IV database corresponding to the patients' first hospitalization. These data encompassed demographic information (age, gender, race), vital signs (heart rate, temperature, respiratory rate, oxygen saturation), comorbidities (hypertension, diabetes), and severity of disease scores [Sequential Organ Failure Assessment (SOFA) and Oxford Acute Severity of Illness Score (OASIS)]. Our main outcome of interest was the incidence of PH.

2.2.3 statistical analyses

Patients were divided into PH and non-PH groups according to whether PH occurred or not. The Shapiro-Wilk test was used to assess the normality of continuous variables. Depending on the distribution of the data, continuous variables were presented as mean ± standard deviation or median (interquartile range), while categorical variables were represented as proportions. Baseline characteristics between the groups were compared utilizing t-tests, chi-square tests, or Mann-Whitney U tests. To assess the relationship between ten ADs and the risk of PH development during hospitalization, a multivariate

logistic regression analysis was conducted. Odds ratios (OR) and their 95% confidence intervals (CI) were computed to measure the influence of ADs on PH. Adjustments were made in the model for potential confounders, including demographic details (age, gender, race), vital signs (heart rate, temperature, respiratory rate, oxygen saturation), comorbidities (hypertension, diabetes), and disease severity scores (SOFA, OASIS). The analysis was carried out using R software version 4.1.3 and SPSS version 22.0 (IBM SPSS Statistics, Armonk, NY, USA). A p-value below 0.05 was considered statistically significant.

2.3 Post-Gwas analysis:

2.3.1 LDSC and SUPERGNOVA

2.3.1.1Study design

We employ linkage disequilibrium score regression (LDSC) as a crucial method for estimating genetic correlations across multiple traits or diseases[24, 25]. To achieve this, we utilized pre-computed LD scores derived from the 1000 Genomes Project. These scores are specifically calculated for SNPs within the HapMap 3 SNP set, excluding those inconsistent with the reference panel. By leveraging summary GWAS statistics alongside LD scores, we applied LDSC to compute heritability for individual traits and ascertain genetic correlations between pairs of traits. In addition, SUPER Genetic covariance analyzer (SUPERGNOVA)was used to calculate localized genetic correlations between T1DM or PBC and PAH. Notably, LDSC yields precise estimates even in scenarios where the test statistic might be inflated due to polygenicity.

2.3.2MAGMA

2.3.2.1 Study Design

As a sensitivity analysis for LDSC, we conducted tissue-specific enrichment and gene set analyses using Multimarker Analysis of GenoMic Annotation (MAGMA)[26]. Initially, gene-level association analyses utilized aggregated data from genome-wide association studies (GWAS), estimating gene-phenotype associations by averaging SNP p-values proximal to target genes. Subsequently, gene set enrichment analyses (MSigDB_20231Hs_MAGMA). Finally, to assess the tissue specificity of the phenotype, we employed MAGMA for gene characterization.

2.3.3Local genetic correlation analysis

ρ-HESS

Heritability estimation using Heritability Estimation from Summary Statistics(ρ-HESS) is a method employed to estimate local SNP heritability and genetic correlations[27]. In this study, we applied ρ-HESS to investigate potential genetic correlations between two autoimmune diseases (ADs), PAH and ADs, and PAH within genetically independent regions of the genome. We computed local SNP heritability for each disease and determined their genetic correlations using the 1000 Genomes Project as a reference dataset, accessible via the ρ-HESS webpage. To account for multiple comparisons, we applied a Bonferroni correction (0.05/ the number of regions) to ensure statistical rigor[27].

2.3.4 Colocalization analysis

We utilized the coloc.abf function from the coloc R package (version 5.1.0) with its default prior to perform colocalization analysis (COLOC). This analysis aimed to calculate the posterior probability that SNPs within significant bivariate loci were either independent (H3) or shared the same correlated variant (H4). The genomic coordinates for co-localization were determined as the boundary reflecting local genetic correlation between the two phenotypes.

2.3.5 PLACO: pleiotropic analysis under composite null hypothesis

We initially extracted disease data relevant to sets of paired trait alliances

exhibiting notable genetic correlations or overlap from the GWAS database. Subsequently, we employed polytomy analysis under the composite null hypothesis (PLACO)[28] to identify potential polytomous single-nucleotide variants (SNVs). SNVs with a PLACO *FDR*-value $< 5 \times 10^{-8}$ were deemed statistically significant.

2.3.6 Summary-data-based Mendelian randomization (SMR) and cis-MR

We employed Summary-data-based Mendelian randomization (SMR) to identify potential functional genes implicated in the statistical associations of PAH and ADs[29]. SMR integrates summary statistics from GWAS and eQTL/pQTL studies within the MR framework to assess the relationship between gene expression and the target phenotype. Analysis was conducted across tissues showing significant SNP-heritability enrichment for both PAH and ADs. Genome-wide significant SNPs were utilized as instrumental variables, and the Heterogeneity in Dependent Instruments (HEIDI) test was applied to assess linkage disequilibrium in the observed associations. SMR further employed the HEIDI-outlier test to discern causality or pleiotropy from linkage. In addition, we include candidate genes with positive SMR analysis results from all eQTL and pQTL sources in the cis-MR analysis. To be selected as candidate genes for cis-MR validation, both autoimmune diseases and pulmonary arterial hypertension must satisfy the following criteria: $P_{(SMR)} < 0.05$, $P_{(HEDI)} > 0.05$, and Total loci ≥ 2[**Figure9**].

2.3.7 FOCUS

We employed fine-mapping of causal gene sets (FOCUS) to compute the posterior inclusion probability (PIP) for each gene within significant bivariate loci[30]. Multi-tissue gene expression weights were derived from the GWAS database, with LD serving as the reference genotypes. Specifically, whole blood cells and lung tissues were chosen as representative tissues. The multi-tissue panel integrated GTEx weights from PrediXcan with weights generated by several studies including the Metabolic Syndrome in Men Study (METSIM), the Dutch Twin Registry (NTR), the Young Finns Study (YFS), and software from the Common Mind Consortium (CMC). Default parameters and settings of FOCUS were uniformly applied across all analyses.

Supplemental References

1. Emdin CA, Khera AV, Kathiresan S. Mendelian randomization. *Jama*. 2017;318:1925-1926
2. Skrivankova VW, Richmond RC, Woolf BAR, Davies NM, Swanson SA, VanderWeele TJ, et al. Strengthening the reporting of observational studies in epidemiology using mendelian randomisation (strobe-mr): Explanation and elaboration. *BMJ (Clinical research ed.)*. 2021;375:n2233
3. Bentham J, Morris DL, Graham DSC, Pinder CL, Tombleson P, Behrens TW, et al. Genetic association analyses implicate aberrant regulation of innate and adaptive immunity genes in the pathogenesis of systemic lupus erythematosus. *Nat Genet*. 2015;47:1457-1464
4. Okada Y, Wu D, Trynka G, Raj T, Terao C, Ikari K, et al. Genetics of rheumatoid arthritis contributes to biology and drug discovery. *Nature*. 2014;506:376-381
5. de Lange KM, Moutsianas L, Lee JC, Lamb CA, Luo Y, Kennedy NA, et al. Genome-wide association study implicates immune activation of multiple integrin genes in inflammatory bowel disease. *Nat Genet*. 2017;49:256-261
6. Sakaue S, Kanai M, Tanigawa Y, Karjalainen J, Kurki M, Koshiba S, et al. A cross-population atlas of genetic associations for 220 human phenotypes. *Nat Genet*. 2021;53:1415-1424
7. Inshaw JRJ, Sidore C, Cucca F, Stefana MI, Crouch DJM, McCarthy MI, et al. Analysis of overlapping genetic association in type 1 and type 2 diabetes. *Diabetologia*. 2021;64:1342-1347
8. Dubois PC, Trynka G, Franke L, Hunt KA, Romanos J, Curtotti A, et al. Multiple common variants for celiac disease influencing immune gene expression. *Nat Genet*. 2010;42:295-302
9. Stuart PE, Tsoi LC, Nair RP, Ghosh M, Kabra M, Shaiq PA, et al. Transethnic analysis of psoriasis susceptibility in south asians and europeans enhances fine-mapping in the mhc and genomewide. *HGG Adv*. 2022;3
10. Cortes A, Hadler J, Pointon JP, Robinson PC, Karaderi T, Leo P, et al. Identification of multiple risk variants for ankylosing spondylitis through high-density genotyping of immune-related loci. *Nat Genet*. 2013;45:730-738
11. Multiple sclerosis genomic map implicates peripheral immune cells and microglia in susceptibility. *Science*. 2019;365
12. Cordell HJ, Fryett JJ, Ueno K, Darlay R, Aiba Y, Hitomi Y, et al. An international genome-wide meta-analysis of primary biliary cholangitis: Novel risk loci and candidate drugs. *J Hepatol*. 2021;75:572-581
13. Scepanovic P, Alanio C, Hammer C, Hodel F, Bergstedt J, Patin E, et al. Human genetic variants and age are the strongest predictors of humoral immune responses to common pathogens and vaccines. *Genome Med*. 2018;10:59
14. Suhre K, Arnold M, Bhagwat AM, Cotton RJ, Engelke R, Raffler J, et al. Connecting genetic risk to disease end points through the human blood plasma proteome. *Nat Commun*. 2017;8:14357
15. Rhodes CJ, Batai K, Bleda M, Haimel M, Southgate L, Germain M, et al. Genetic determinants of risk in pulmonary arterial hypertension: International genome-wide association studies and meta-analysis. *Lancet Respir Med*. 2019;7:227-238
16. Xie Z, Zhou H, Qiu Z, Fan Z, Yang W, Zhang J, et al. Association between 23 drugs and parkinson's disease: A two-sample mendelian randomization study. *Brain Behav*. 2023;13:e3225

17. Gao X, Meng LX, Ma KL, Liang J, Wang H, Gao Q, et al. The bidirectional causal relationships of insomnia with five major psychiatric disorders: A mendelian randomization study. *Eur Psychiatry*. 2019;60:79-85
18. Cao Z, Wu Y, Li Q, Li Y, Wu J. A causal relationship between childhood obesity and risk of osteoarthritis: Results from a two-sample mendelian randomization analysis. *Ann Med*. 2022;54:1636-1645
19. Deitiker P, Atassi MZ. Mhc genes linked to autoimmune disease. *Crit Rev Immunol*. 2015;35:203-251
20. Wang L, Wang FS, Gershwin ME. Human autoimmune diseases: A comprehensive update. *J Intern Med*. 2015;278:369-395
21. Verbanck M, Chen CY, Neale B, Do R. Detection of widespread horizontal pleiotropy in causal relationships inferred from mendelian randomization between complex traits and diseases. *Nat Genet*. 2018;50:693-698
22. Johnson AEW, Bulgarelli L, Shen L, Gayles A, Shammout A, Horng S, et al. Mimic-iv, a freely accessible electronic health record dataset. *Sci Data*. 2023;10:1

eFigure 1.

Exposure	Source	SNPs	OR	LCI	UCI	P-value
Ankylosing spondylitis	FinnGen Consortium	33	0.941	0.888	0.997	0.040
	MRC-IEU	18	0.958	0.567	1.618	0.871
Celiac disease	Gwas Catalog	35	1.071	1.007	1.138	0.028
	MRC-IEU	12	1.133	1.017	1.263	0.024
Crohn's disease	FinnGen Consortium	21	0.963	0.871	1.066	0.470
	MRC-IEU	88	1.034	0.977	1.094	0.250
Multiple sclerosis	FinnGen Consortium	10	0.965	0.846	1.100	0.590
	MRC-IEU	60	0.948	0.878	1.024	0.175
Primary biliary cholangitis	Gwas Catalog	19	1.144	1.071	1.222	7.06E-05
	MRC-IEU	34	1.100	1.034	1.171	0.003
Psoriasis	FinnGen Consortium	4	1.022	0.786	1.330	0.869
	MRC-IEU	47	1.034	0.953	1.122	0.420
Rheumatoid arthritis	FinnGen Consortium	4	1.276	1.016	1.603	0.036
	MRC-IEU	43	1.198	1.090	1.317	1.77E-04
Systemic lupus erythematosus	FinnGen Consortium	15	0.966	0.885	1.055	0.440
	MRC-IEU	38	0.982	0.937	1.029	0.445
Type 1 diabetes	FinnGen Consortium	4	1.250	1.021	1.531	0.031
	MRC-IEU	26	1.139	1.048	1.238	0.002
Ulcerative colitis	FinnGen Consortium	6	0.981	0.807	1.193	0.849
	MRC-IEU	6	0.978	0.782	1.224	0.848

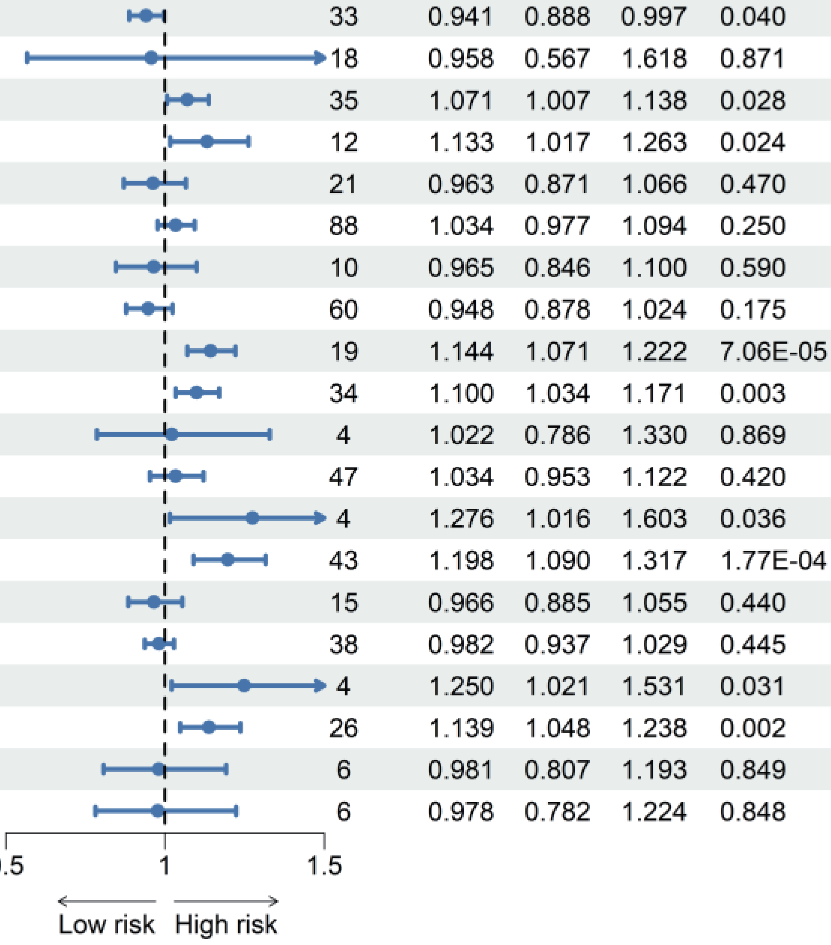

eFigure 2.

Exposure	Source	SNPs	OR	LCI	UCI	P-value
Ankylosing spondylitis	FinnGen Consortium	33	0.941	0.888	0.997	0.040
	MRC-IEU	26	0.791	0.589	1.063	0.120
Celiac disease	FinnGen Consortium	36	1.074	1.012	1.139	0.019
	MRC-IEU	12	1.133	1.017	1.263	0.024
Crohn's disease	FinnGen Consortium	21	0.963	0.871	1.066	0.470
	MRC-IEU	88	1.034	0.977	1.094	0.250
Multiple sclerosis	FinnGen Consortium	13	1.038	0.956	1.128	0.369
	MRC-IEU	61	0.947	0.879	1.021	0.155
Primary biliary cholangitis (PBC)	FinnGen Consortium	20	1.143	1.072	1.220	4.70E-05
	MRC-IEU	39	1.131	1.064	1.203	8.30E-05
Psoriasis	FinnGen Consortium	7	1.047	0.850	1.289	0.666
	MRC-IEU	56	0.985	0.921	1.054	0.663
Rheumatoid arthritis	FinnGen Consortium	4	1.276	1.016	1.603	0.036
	MRC-IEU	49	1.160	1.062	1.268	0.001
Systemic lupus erythematosus	FinnGen Consortium	16	0.971	0.890	1.060	0.511
	MRC-IEU	40	0.981	0.936	1.027	0.407
Type 1 diabetes	FinnGen Consortium	16	1.107	1.016	1.206	0.020
	MRC-IEU	36	1.049	1.012	1.086	0.008
Ulcerative colitis	FinnGen Consortium	6	0.981	0.807	1.193	0.849
	MRC-IEU	6	0.978	0.782	1.224	0.848

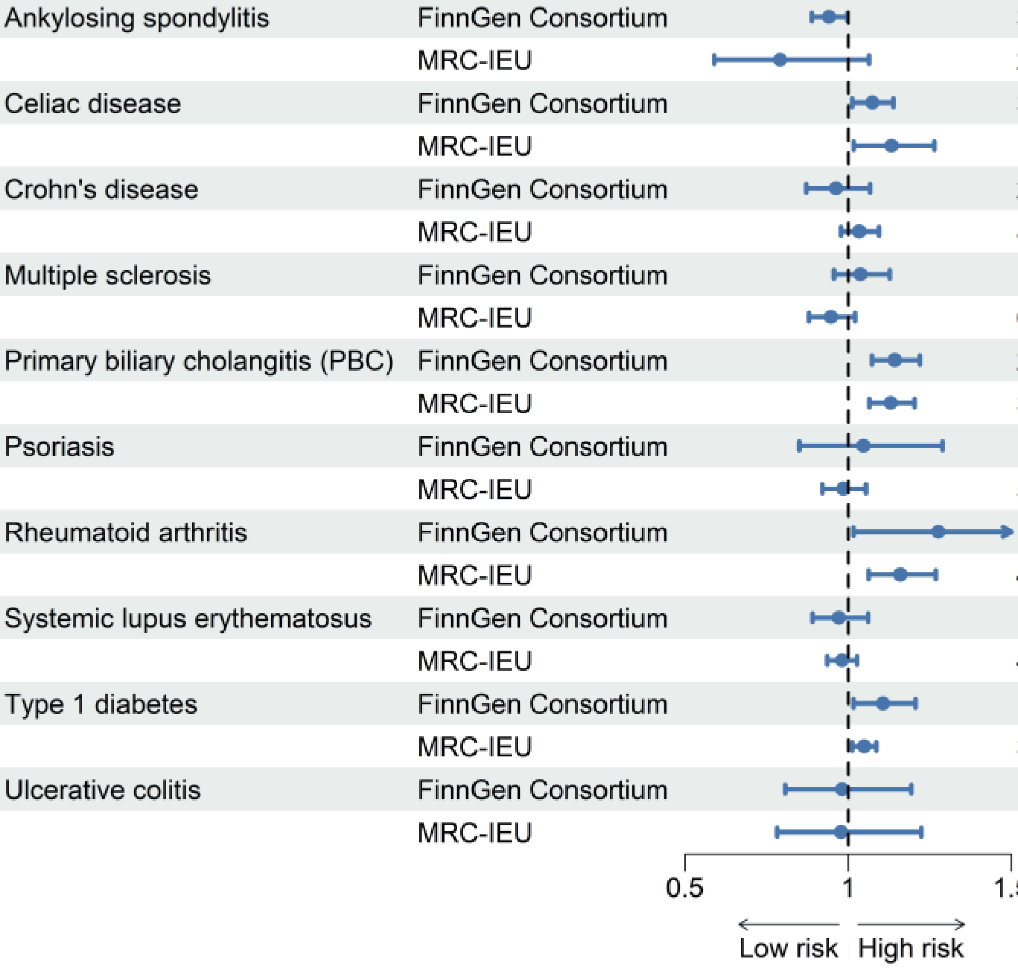

eFigure 3.

Exposure	Outcome	SNPs	OR	LCI	UCI	P-value
Pulmonary arterial hypertension	Ankylosing spondylitis	10	1.017	0.998	1.036	0.075
Pulmonary arterial hypertension	Coeliac disease	5	0.973	0.785	1.208	0.807
Pulmonary arterial hypertension	Crohns disease	16	1.005	0.971	1.040	0.797
Pulmonary arterial hypertension	Multiple sclerosis	12	0.961	0.916	1.009	0.107
Pulmonary arterial hypertension	Primary biliary cholangitis	7	1.020	0.945	1.102	0.609
Pulmonary arterial hypertension	Psoriasis	20	1.000	0.999	1.001	0.223
Pulmonary arterial hypertension	Rheumatoid arthritis	15	1.000	0.988	1.012	0.944
Pulmonary arterial hypertension	Systemic lupus erythematosus	15	1.090	1.014	1.171	0.019
Pulmonary arterial hypertension	Type 1 diabetes	17	1.047	0.992	1.105	0.098
Pulmonary arterial hypertension	Ulcerative colitis	19	0.997	0.969	1.025	0.817

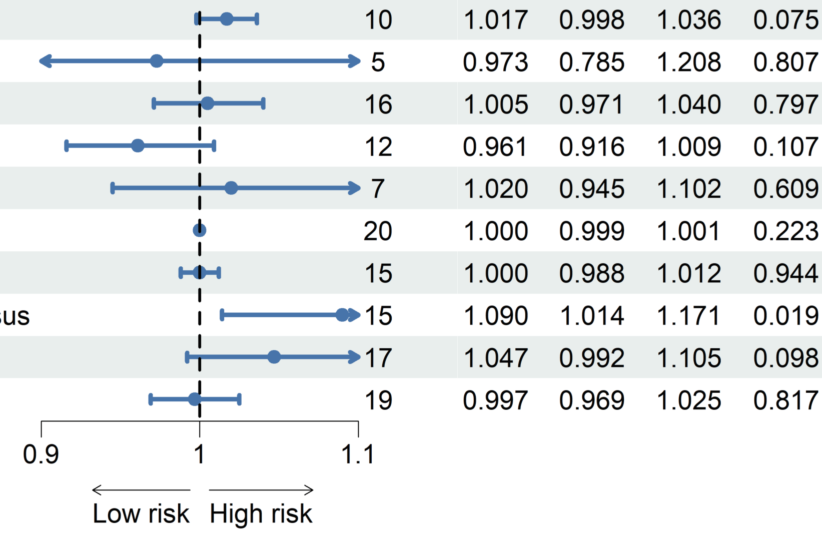

eFigure 4.

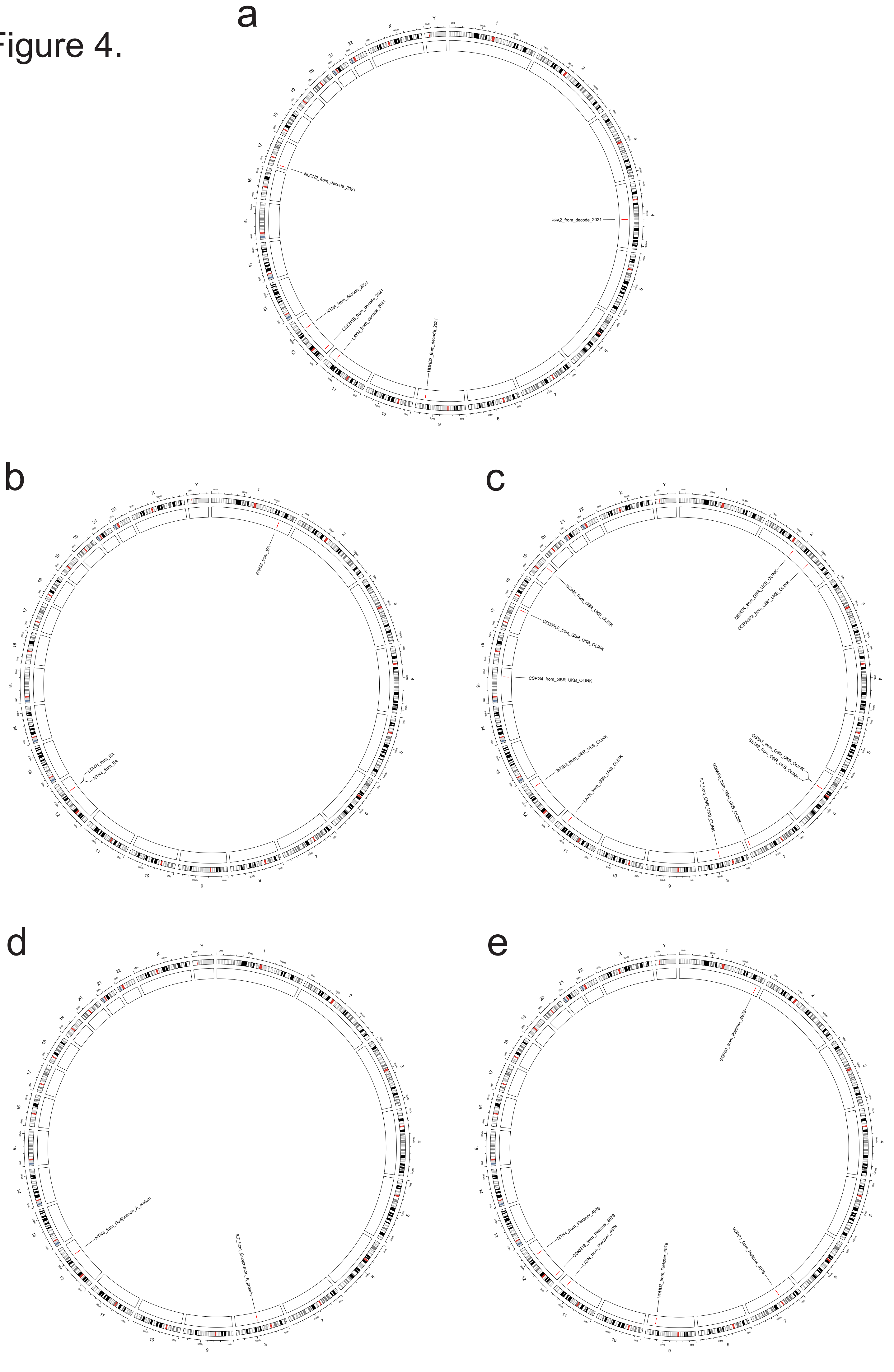
a
NLGN2_from_decode_2021
PPA2_from_decode_2021
NTN4_from_decode_2021
CDKN1B_from_decode_2021
LAYN_from_decode_2021
HDHD3_from_decode_2021
b
FAIM3_from_EA
LTA4H_from_EA
NTN4_from_EA
c
BCAM_from_GBR_UKB_OLINK
CD300LF_from_GBR_UKB_OLINK
CSPG4_from_GBR_UKB_OLINK
MERTK_from_GBR_UKB_OLINK
GORASP2_from_GBR_UKB_OLINK
SH2B3_from_GBR_UKB_OLINK
LAYN_from_GBR_UKB_OLINK
IL7_from_GBR_UKB_OLINK
GIMAP8_from_GBR_UKB_OLINK
GSTA1_from_GBR_UKB_OLINK
GSTA3_from_GBR_UKB_OLINK
d
NTN4_from_Gudjonsson_A_protein
IL7_from_Gudjonsson_A_protein
e
GGPS1_from_Pietzner_4979
NTN4_from_Pietzner_4979
CDKN1B_from_Pietzner_4979
LAYN_from_Pietzner_4979
HDHD3_from_Pietzner_4979
VOPP1_from_Pietzner_4979

eFigure 5.

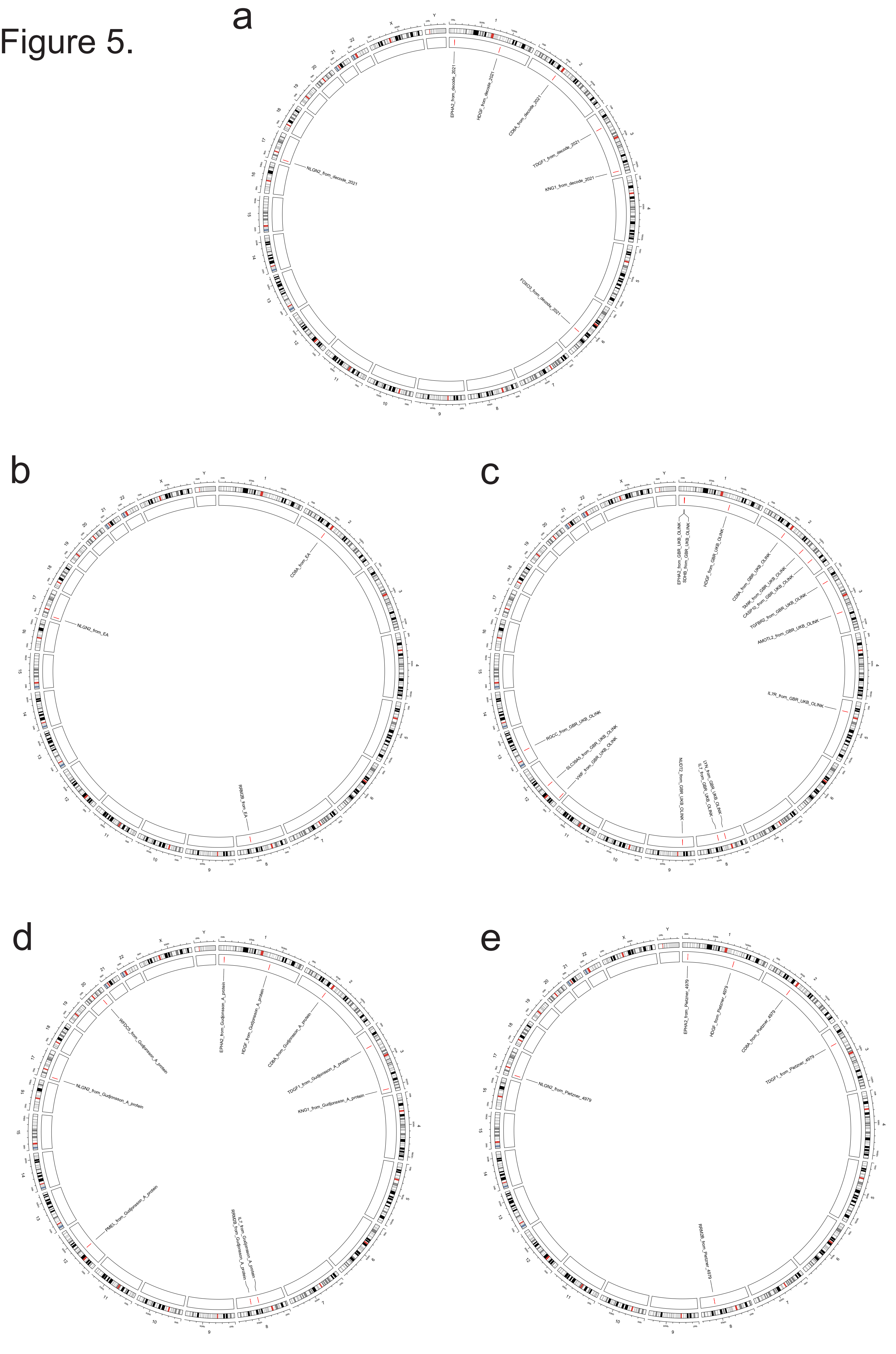
a
EPHA2_from_decode_2021
HDGF_from_decode_2021
CD8A_from_decode_2021
TDGF1_from_decode_2021
KNG1_from_decode_2021
FOXO3_from_decode_2021
NLGN2_from_decode_2021
b
CD8A_from_EA
NLGN2_from_EA
RRM2B_from_EA
c
EPHA2_from_GBR_UKB_OLINK
SDHB_from_GBR_UKB_OLINK
HDGF_from_GBR_UKB_OLINK
CD8A_from_GBR_UKB_OLINK
TANK_from_GBR_UKB_OLINK
CASP10_from_GBR_UKB_OLINK
TGFBR2_from_GBR_UKB_OLINK
AMOTL2_from_GBR_UKB_OLINK
IL7R_from_GBR_UKB_OLINK
RGCC_from_GBR_UKB_OLINK
SLC39A5_from_GBR_UKB_OLINK
VWF_from_GBR_UKB_OLINK
NUDT2_from_GBR_UKB_OLINK
LYN_from_GBR_UKB_OLINK
IL7_from_GBR_UKB_OLINK
d
EPHA2_from_Gudjonsson_A_protein
HDGF_from_Gudjonsson_A_protein
CD8A_from_Gudjonsson_A_protein
TDGF1_from_Gudjonsson_A_protein
KNG1_from_Gudjonsson_A_protein
WFDC5_from_Gudjonsson_A_protein
NLGN2_from_Gudjonsson_A_protein
PMEL_from_Gudjonsson_A_protein
IL7_from_Gudjonsson_A_protein
RRM2B_from_Gudjonsson_A_protein
e
EPHA2_from_Pietzner_4979
HDGF_from_Pietzner_4979
CD8A_from_Pietzner_4979
TDGF1_from_Pietzner_4979
NLGN2_from_Pietzner_4979
RRM2B_from_Pietzner_4979

eFigure 6.

a

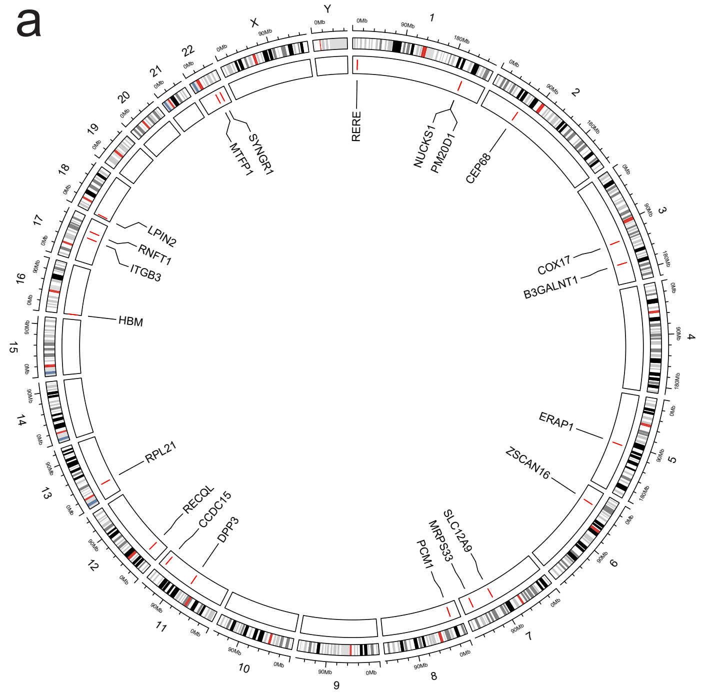

b

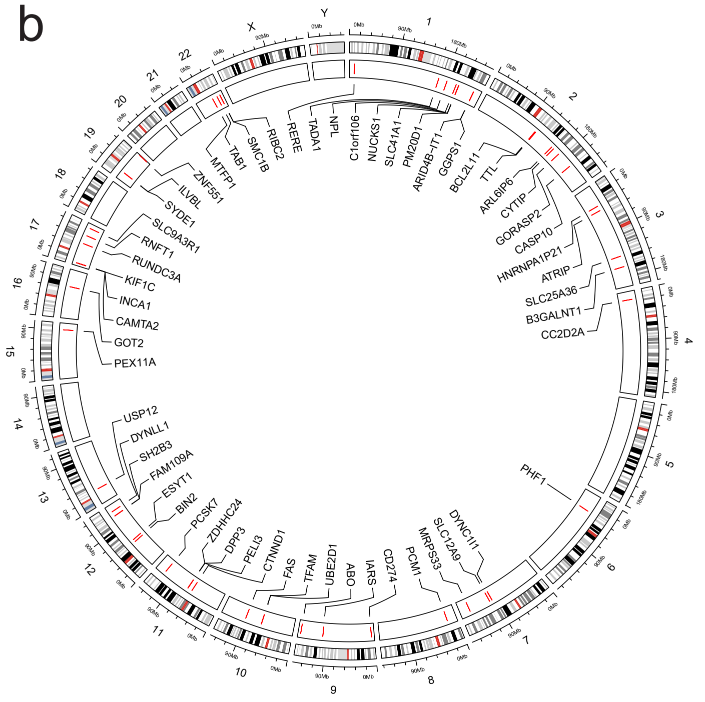

c

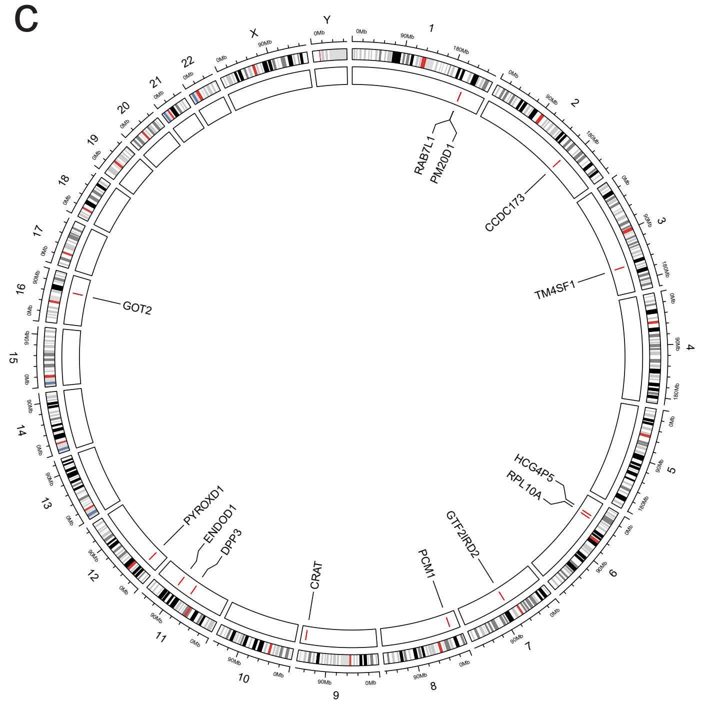

d

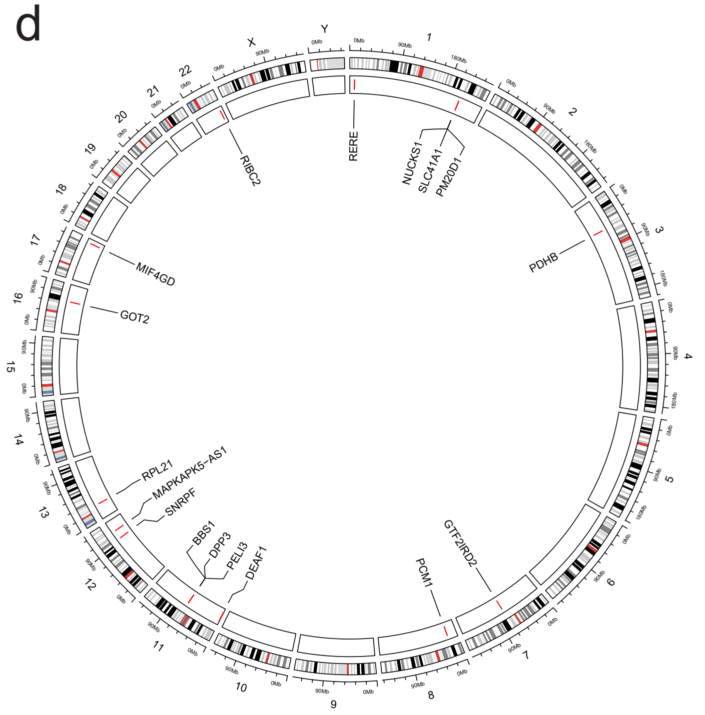

eFigure 7.

a

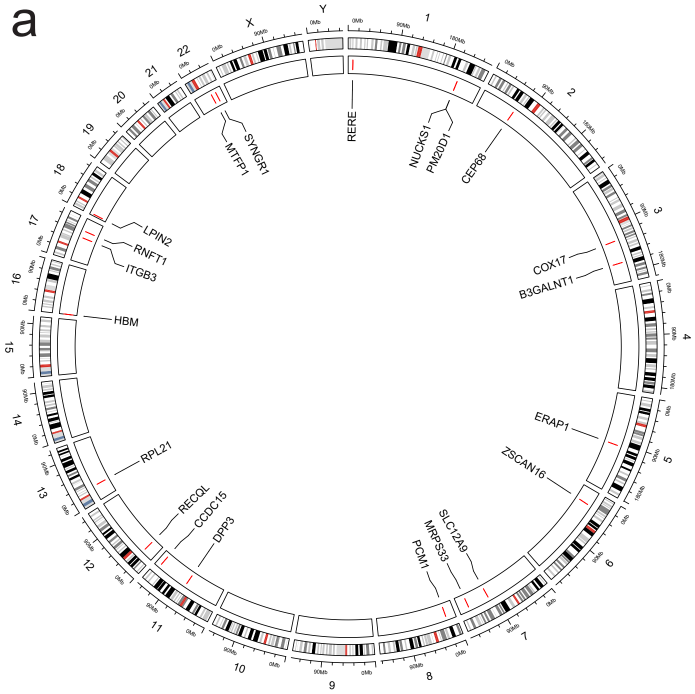

b

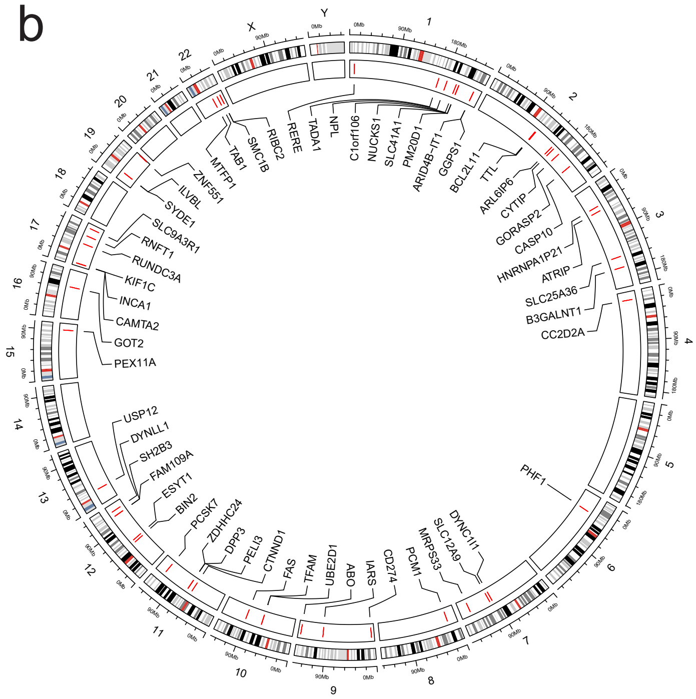

c

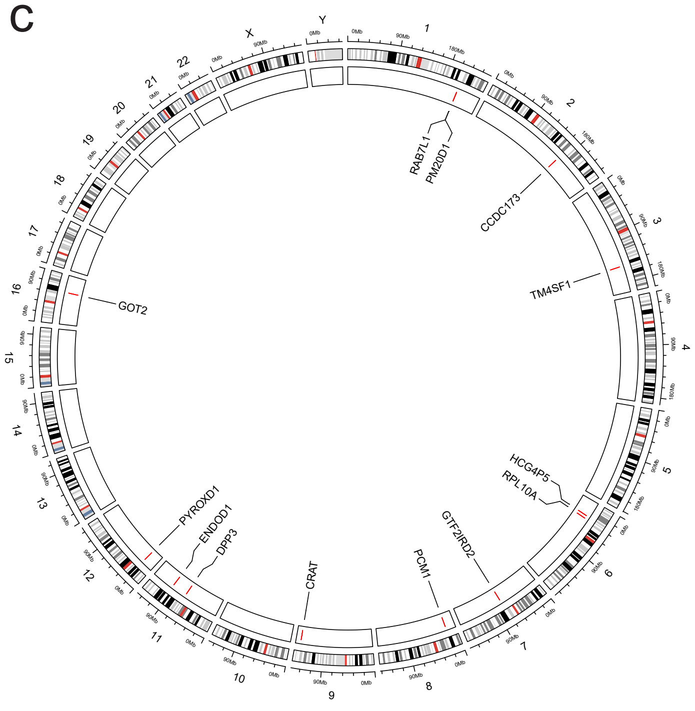

d

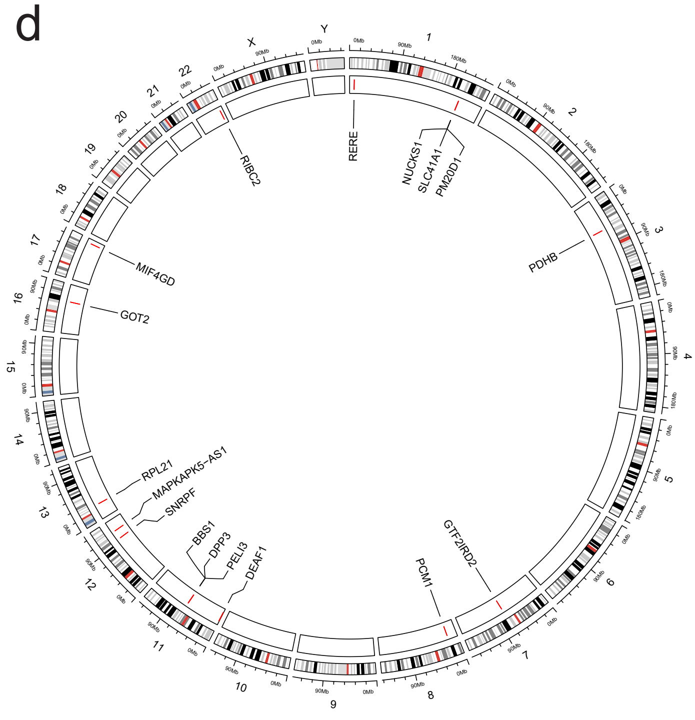

eFigure 1: Forest plot to visualize the causal effect of autoimmune diseases (non-MHC loci SNPs) on PAH using the inverse variance-weighted method.CI: 95% confidence interval. OR, odds ratio.

eFigure 2: Forest plot to visualize the causal effect of autoimmune diseases (included-MHC loci SNPs) on PAH using the inverse variance-weighted method. CI: 95% confidence interval. OR, odds ratio.

eFigure 3: Forest plot to visualize the causal effect of PAH and autoimmune diseases using the inverse variance-weighted method. CI: 95% confidence interval. OR, odds ratio.

eFigure 4: SMR proteomic analysis between PAH and PBC.

To be selected as candidate genes for Heat Map, both autoimmune diseases and pulmonary arterial hypertension must satisfy the following criteria: $P_{(SMR)} < 0.05$, $P_{(HEDI)} > 0.05$. a:SMR analysis graph for PAH and PBC from decode 2021; b:SMR analysis graph for PAH and PBC from EA;c:SMR analysis graph for PAH and PBC from GBR UKB OLINK; d:SMR analysis graph for PAH and PBC from Gudjonsson A protei 4782; e:SMR analysis graph for PAH and PBC from Pietzner 4979.

eFigure 5:SMR proteomic analysis between PAH and T1DM.

To be selected as candidate genes for Heat Map, both autoimmune diseases and pulmonary arterial hypertension must satisfy the following criteria: $P_{(SMR)} < 0.05$, $P_{(HEDI)} > 0.05$. a:SMR analysis graph for PAH and T1DM from decode 2021; b:SMR analysis graph for PAH and T1DM from EA;c:SMR analysis graph for PAH and T1DM from GBR UKB OLINK; d:SMR analysis graph for PAH and T1DM from Gudjonsson A protei 4782; e:SMR analysis graph for PAH and T1DM from Pietzner 4979.

eFigure 6: SMR transcriptomic analyses of PAH and PBC.

To be selected as candidate genes for Heat Map, both autoimmune diseases and pulmonary arterial hypertension must satisfy the following criteria: $P_{(SMR)} < 0.05$, $P_{(HEDI)} > 0.05$. a:SMR analysis graph for PAH and PBC from CAGE; b:SMR analysis graph for PAH and PBC from eQTLGen;c:SMR analysis graph for PAH and PBC from Lung_GTEx_V8; d:SMR analysis graph for PAH and PBC from Whole_Blood_GTEx_V8.

eFigure 7: SMR transcriptomic analyses of PAH and T1DM.

To be selected as candidate genes for Heat Map, both autoimmune diseases and pulmonary arterial hypertension must satisfy the following criteria: $P_{(SMR)} < 0.05$, $P_{(HEDI)} > 0.05$. a:SMR analysis graph for PAH and T1DM from CAGE; b:SMR analysis graph for PAH andT1DM from eQTLGen;c:SMR analysis graph for PAH and T1DM from Lung_GTEx_V8; d:SMR analysis graph for PAH and T1DM from Whole_Blood_GTEx_V8.
